# SYTL4 downregulates microtubule stability and confers paclitaxel resistance in triple-negative breast cancer

**DOI:** 10.7150/thno.45207

**Published:** 2020-08-29

**Authors:** Xi-Yu Liu, Wang Jiang, Ding Ma, Li-Ping Ge, Yun-Song Yang, Zong-Chao Gou, Xiao-En Xu, Zhi-Ming Shao, Yi-Zhou Jiang

**Affiliations:** 1 Key Laboratory of Breast Cancer in Shanghai, Department of Breast Surgery, Fudan University Shanghai Cancer Center; Department of Oncology, Shanghai Medical College, Fudan University, Shanghai 200032, China.; 2 University of Chinese Academy of Sciences, Beijing 100049, China.

**Keywords:** Triple-negative breast cancer, paclitaxel resistance, SYTL4, microtubule polymerization

## Abstract

**Background:** Taxanes are frontline chemotherapeutic drugs for patients with triple-negative breast cancer (TNBC); however, chemoresistance reduces their effectiveness. We hypothesized that the molecular profiling of tumor samples before and after neoadjuvant chemotherapy (NAC) would help identify genes associated with drug resistance.

**Methods:** We sequenced 10 samples by RNA-seq from 8 NAC patients with TNBC: 3 patients with a pathologic complete response (pCR) and the other 5 with non-pCR. Differentially expressed genes that predicted chemotherapy response were selected for *in vitro* functional screening via a small-scale siRNAs pool. The clinical and functional significance of the gene of interest in TNBC was further investigated *in vitro* and *in vivo*, and biochemical assays and imaging analysis were applied to study the mechanisms.

**Results:** Synaptotagmin-like 4 (SYTL4), a Rab effector in vesicle transport, was identified as a leading functional candidate. High SYTL4 expression indicated a poor prognosis in multiple TNBC cohorts, specifically in taxane-treated TNBCs. SYTL4 was identified as a novel chemoresistant gene as validated in TNBC cells, a mouse model and patient-derived organoids. Mechanistically, downregulating SYTL4 stabilized the microtubule network and slowed down microtubule growth rate. Furthermore, SYTL4 colocalized with microtubules and interacted with microtubules through its middle region containing the linker and C2A domain. Finally, we found that SYTL4 was able to bind microtubules and inhibit the *in vitro* microtubule polymerization.

**Conclusion:** SYTL4 is a novel chemoresistant gene in TNBC and its upregulation indicates poor prognosis in taxane-treated TNBC. Further, SYTL4 directly binds microtubules and decreases microtubule stability.

## Introduction

Breast cancer is the most common cancer that threatens the health of women worldwide 1. Triple-negative breast cancer (TNBC), defined by the lack of expression of the estrogen receptor (ER), the progesterone receptor (PR) and the human epidermal growth factor receptor (HER2) 2, comprises 12-20% of breast cancers and is characterized by poor prognosis, early metastasis, and aggressive tumor behavior 3, 4. Our recent study classified TNBC into four different subtypes based on transcriptome profiles, namely, luminal androgen receptor (LAR), immunomodulatory (IM), basal-like immune-suppressed (BLIS), and mesenchymal (MES), suggesting that TNBC itself is heterogeneous. Among these subtypes, the MES subtype exhibits the poorest prognosis but lacks distinctive genomic alterations 5, 6. New potential therapeutic targets are emerging for TNBCs, such as folate receptor alpha (FRα) 7, and the supernumerary centrosomes 8.

Neoadjuvant chemotherapy (NAC) based on paclitaxel has served as a standard treatment for many patients with TNBC, especially for locally advanced disease 9. Although a proportion of patients with TNBC show an excellent pathologic complete response (pCR), 30-50% develop resistance, leading to a poor prognosis 10. Discovering novel oncogenes driving TNBC progression is required for developing new therapeutic targets for TNBC. Several studies have compared transcriptomic differences in pre-NAC samples between responders and nonresponders and established predictive signatures to drug response 11-14. Furthermore, profiling residual tumors after NAC can identify genes associated with drug resistance via bulk sequencing 15-17 or single-cell sequencing 18. On the other side, overexpression of drug-efflux pumps 19, alterations in microtubule-associated proteins 20, 21, aberrant cancer stem cell signaling 22, 23, and activated PI3K/Akt pathway 24, 25, etc., have been reported to be associated with chemoresistance.

In this study, we sequenced 10 TNBC tumor samples from 8 patients who received taxane-containing NAC in Fudan University Shanghai Cancer Center (FUSCC). Differentially expressed genes were further screened by *in vitro* siRNA to identify paclitaxel resistance-associated genes.

## Materials and Methods

### RNA-sequencing and bioinformatic analysis

For tumor RNA-sequencing (RNA-seq), ten freshly frozen tissues were collected from eight patients with TNBC who received neoadjuvant paclitaxel and carboplatin at FUSCC. Among these ten samples, eight were obtained by core needle biopsy as pre-NAC baseline samples, and two were surgically resected tumors as matched post-NAC residual samples from two patients with progressed disease (PD). Core needle biopsies contained 70-90% pure tumor cells with minimal stromal contamination. RNA was isolated using the RNeasy mini kit (Qiagen, Germany) and sequenced by RNA-seq on an Illumina HiSeq 2500 platform by GENESEQ (Nanjing, China). Reads were aligned to the GRCh37 genome using STAR 26. Gene expression levels were quantified as transcripts per kilobase million (TPM) using RNA-Seq by Expectation-Maximization (RSEM) 27. Differentially expressed genes (DEG) were identified using R package 'limma'.

### Gene Set Enrichment Analysis and pathway analysis

We analyzed the enriched pathways of candidate genes in the Kyoto Encyclopedia of Genes and Genomes (KEGG) database (http://www.genome.jp/kegg/) 28. The top 20 enriched KEGG pathways were plotted in a bubble plot. “Rich factor” means the ratio of the number of called genes to the background number annotated in a certain pathway. The greater the “Rich factor”, the greater is the degree of enrichment.

We evaluated the differences in expression between the high and low SYTL4-expression groups on a set of 50 hallmark signatures downloaded from MSigDB 29, 30 using the Gene Set Enrichment Analysis (GSEA) software (GSEA 2.2.1, http://software.broadinstitute.org/gsea/).

### Cell cultures

TNBC cell lines (MDA-MB-231, Hs578T, HCC1143, HCC1937, HCC1599, MDA-MB-157, BT-20, MDA-MB-436, HCC38 and HCC70) and 293T cells were obtained from the American Type Culture Collection (Manassas, VA, USA) and maintained in complete DMEM growth medium containing 10% fetal bovine serum (FBS) (Gibco, USA), 2 mM L-glutamine, 100 IU/mL penicillin, and 100 μg/mL streptomycin 31. Liquid nitrogen stocks were created upon receipt, and cells were maintained until the start of each study. Cells were used for no more than 10 passages after being thawed. All cell lines tested negative for mycoplasma contamination.

### siRNA pool assay

The custom small interfering RNA (siRNA) pool was synthesized with three siRNA duplexes for each gene (RiboBio, China). In total, the pool contains 90 targets for 30 genes and one nontargeting siRNA. To compare the effect of a drug on siRNA-transfected cells with the effect on cells transfected with the nontargeting siRNA, the “sensitization index” (SI) 32 was used: SI = (Rc/Cc)*(Cd/Cc) - Rd/Cc, where the symbols represent the absorbance measured after incubating cells with CCK-8 reagent: **Rc** siRNA without drug; **Cc** nontargeting siRNA without drug; **Cd** nontargeting siRNA with drug; **Rd** siRNA without drug. An average SI was calculated for each siRNA across triplicates following paclitaxel treatment. An individual siRNA target with an average SI > 0.1 was considered a synergistic effector with paclitaxel.

### Transient knockdown

A mixture of 50 μL of Opti-MEM (Invitrogen, USA), 0.3 μL of Lipofectamine RNAiMAX Transfection Reagent (Thermo Fisher Scientific, USA) and 50 nM individual siRNA was preincubated in triplicate in 96-well plates. Cells were seeded in 100 μL of antibiotic-free DMEM containing 10% FBS (3000 cells/well). Target sequences of siRNA for transient knocking down SYTL4 were as follows: siRNA1: 5'-GCAGCATGATGAGCATCTA-3'; siRNA2: 5'-GTCTGGTTGTCCATGTGAA-3'; siRNA3: 5'-GCTGGCCTATGCTGATGAA-3'; siRNA4: 5'-GGATATGGAAGAGGAAGAA-3'; siRNA5: 5'-GGATTTGATTCTCAGTGTT-3'. Scrambled siRNA was used as a control. The siRNA transfection was conducted according to the manufacturer's protocol.

### Plasmids, lentivirus infection and generation of stable cell lines

Target sequences of short hairpin RNA (shRNA) for stably knocking down SYTL4 were as follows: shNC: 5'-TTCTCCGAACGTGTCACGT-3'; shSYTL4-1: 5'-TCCCTTTACATGGAAAGAT-3'; shSYTL4-2: 5'-CCAAGGAAATAGAGTTGAA-3'.

Lentiviral plasmids containing the fragment of SYTL4, SYTL4-GFP, or three domains of SYTL4 fused with GFP at their C-terminus (D1/D2/D3-GFP) were constructed by inserting corresponding PCR product into the pCDH-CMV vector between BamHI and EcoRI sites. Constructs were examined by Sanger sequencing and selected for further experiments.

Using Lipofectamine 2000 transfection reagent (Thermo Fisher Scientific, USA), 293T cells were co-transfected with lentivirus vectors and packaging vectors (psPAX2 and pMD2.G). Viral supernatants were collected and filtered after 48 h. Cells infected by the lentivirus were subjected to puromycin selection. Alterations in the expression level were verified by western blot analysis.

### Western blot analysis

Western blotting was performed as described previously 31. Briefly, equal amounts of protein samples were resolved by SDS-PAGE and transferred to PVDF membranes (Roche, Switzerland). Blocking was performed with 5% skim milk, and blotting was performed with primary antibodies and secondary HRP-conjugated antibodies as indicated. The signal was detected by enhanced chemiluminescence substrate (Pierce Biotechnology, USA) under Tanon 4200. For quantification, the intensity of the western blot band was analyzed using Fiji (National Institutes of Health, USA).

### Cell proliferation assay

For half maximal inhibitory concentration (IC50) determination, cells (3 × 10^3^ per well) were seeded in 96-well plates. After 24 h, the cells were treated with the indicated concentrations of drug or DMSO for an additional 3 days. Cytotoxicity was determined using the CCK-8 assay as described previously 31. Briefly, the CCK-8 was prediluted with complete DMEM growth medium at 1:10, and 100 μL solution was added to each well. After incubation in 37 °C in the dark for 2-3 h, O.D values of plates were measured using a spectrometer at 450 nm. The IC50 was determined according to a dose vs. response curve by GraphPad Prism 6.0.

For the *in vitro* cell growth assay, cells were seeded in 96-well plates and monitored using the IncuCyte ZOOM System (Essen BioScience, Germany). Images were captured at 12 h intervals from four separate regions per well. The relative survival rate was calculated by dividing the confluence of paclitaxel-treated cells by the confluence of DMSO-treated cells (%).

### Colony-formation assay

Briefly, 3000 cells were plated in triplicate in 1 mL of complete growth medium in a 24-well plate. The following day, paclitaxel or an equal volume of DMSO was added to the medium. After 2 weeks, the colonies were stained with 0.5% methylene blue in 50% ethanol. Colonies larger than 9 pixels^2^ (50 cells) were counted by the particle analysis plugin in Fiji (National Institutes of Health, USA). The relative colony-forming efficiency was calculated by dividing the number of paclitaxel-treated colonies by the number of DMSO-treated colonies (%).

### Patient-derived organoid culture

Histologically diagnosed TNBC tissues were obtained from freshly resected specimens during surgery at FUSCC. Patients were informed before the surgery and agreed by written consent to tissue collection. Tissue processing, organoid culture and drug sensitivity test were performed as described previously 33, 34. Tumors were processed immediately upon receipt and cultured as described below. These untrypsinized organoids were embedded in 30 or 80 μL Matrigel depending on the tumor volume. After solidification of the Matrigel-cell solution in 24-well plates, breast cancer organoid medium was added immediately and changed every 4 days. Organoids were harvested for qRT-PCR analysis after 2 to 4 weeks. Organoids were trypsinized and passaged approximately every 14-21 d.

For lentiviral transduction in organoids, high titer virus was collected through ultracentrifugation at 50,000 × *g* for 90 min, resuspended with 500 μL of organoid culture medium supplemented with 8 μg/mL polybrene and stored at -80 °C as described previously 35. Organoids were split into new wells to obtain small organoids two days before transfection. On the day of transfection, organoids were harvested, trypsinized, resuspended with 20 μL of medium, and mixed with 250 μL of high titer lentivirus. Then, the organoid-virus mixture was incubated in a 48-well plate for 3 hours at 37 °C, centrifuged, washed and seeded in a 48-well plate. Three days later, fresh medium with puromycin (4 μg/mL) was added. Organoids were selected by puromycin for 2 weeks and then were harvested for qRT-PCR analysis after 2-4 weeks.

For the drug sensitivity test, organoids were harvested and diluted to 75 organoids/μL in growth medium. Black, clear-bottom 384-well plates (Corning) were coated with 10 µl of basement membrane extract (BME) before the addition of 30 µl of organoid suspension. Then, six concentrations of paclitaxel, as well as the DMSO control, were added in triplicate. CellTiter-Glo 3D Reagent (Promega) was added after five days, and the plate was agitated on a shaker for 30 min at room temperature. The luminescence was measured with a SpectraMax microplate reader (Molecular Devices). Data were analyzed using GraphPad Prism 8.0, followed by the manual determination of the IC50 values.

### Measurement of tubulin polymerization

Cells were grown in duplicate wells of 24-well plates to 80% confluence and remained untreated or treated overnight with 10 nM paclitaxel for 16 to 18 hours. The tubulin in microtubules was measured as described previously 36. Briefly, the cells were lysed in a microtubule-stabilizing buffer, which contains 20 mM Tris-HCl (pH 6.8), 0.14 M NaCl, 0.5% NP40, 1 mM MgCl_2_, 2 mM EGTA, and 4 μg/mL paclitaxel. The lysates were then centrifuged at 10,000 × *g* for 20 min to obtain the pellet fraction containing microtubules (*P*) and supernatant fraction containing soluble tubulin (*S*). Western blot analysis was performed using the indicated antibodies. The percentage of assembled tubulin was calculated as:


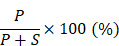


### Tubulin turbidity assay

The microtubule polymerization was monitored by measuring changes in absorbance (340 nm) by a spectrophotometer as described previously 37. Briefly, all of the components of the reactions from the Tubulin Polymerization Assay Kit (Cytoskeleton, USA) were mixed in 96-well plates according to the manufacturer's protocol. After gentle mixing, the absorbances of the reaction mixtures were immediately determined by a spectrophotometer at 37 °C for 60 min.

### Immunofluorescence imaging

Staining and imaging of monolayer cells were performed as described previously 31. In detail, cells grown on glass coverslips were first washed with 37 °C 1×PBS to avoid cold-induced depolymerization of microtubule. Then, 4% formaldehyde (in PBS) was used for fixation for 15 min at room temperature. Next, cells on coverslips were permeabilized by 0.25% TritonX-100, blocked with 5% BSA in 1xPBS, and then incubated with primary antibodies for 1 h at room temperature, which included mouse monoclonal anti-α-tubulin (Proteintech, USA) and rabbit polyclonal anti-SYTL4 (Proteintech, USA). After being incubated with appropriate fluorescent secondary antibodies (Invitrogen, USA) and washed, the nuclei were counterstained with DAPI (Sigma-Aldrich, USA). Finally, the glass coverslips were mounted onto glass slides with Fluoromount aqueous mounting medium (Sigma). Images were acquired and deconvolved using Deltavision Elite (GE, USA) or captured by an Olympus IX53 fluorescence microscope (Olympus Corporation, USA). For super-resolution images, slides were scanned by structured illumination microscopy (SIM) technology by DeltaVision OMX SR (GE Healthcare, USA).

Polymerized microtubules were evaluated by Fiji using the Tubeness plugin. Original images were converted into 8-bit format and followed by Tubeness analysis (sigma = 1.0) to select tubule-like structures. Then, images were converted into 8-bit format again and applied with the same threshold for comparison. Particle analysis was further used to determine the area of the microtubule network to the whole cell. The percentage (%) of polymerized microtubules was represented by the ratio of the area of tubule-like structure to the region inside the cell contour.

### Live cell tracking of EB1 motion

EB1 is a microtubule end-binding protein that tracks the tips of growing microtubules. The EB1-ΔC-GFP construct 38 was synthesized (Genscript) and cloned into a lentiviral vector and then was stably expressed in cells by lentiviral transduction. For real-time tracking of EB1 motion, DeltaVision Elite imaging system was used with a 100× /1.40 NA Plan Apo oil-immersion objective (Olympus), and the CoolSnap HQ2 camera (Photometrics) equipped with the live cell imaging environment control system (Live Cell Instrument). Images were captured in 30 s with 14 intervals and deconvolved by the DeltaVision Elite software. The motion and speed of EB1 were analyzed by the particle tracking function in Imaris 9.0.

### Coimmunoprecipitation

Cells grown in 10-cm dishes were washed once with 1×PBS and lysed with Western/IP lysis buffer (Beyotime, China) supplemented with 0.5 mM PMSF. Following 12,000 × *g* centrifugation at 4 °C for 5 min, the pellet containing the cell debris was removed, and the supernatant was harvested for Co-IP. Anti-GFP nanobody-coated agarose (KT health, China) was used according to the manufacturer's protocol. Briefly, cell lysate supernatant was mixed with anti-GFP nanobody suspension and incubated overnight on a rotator at 4 °C. The agarose beads were pelleted by centrifugation at 2,400 × *g* for 1 min at 4 °C and washed three times with the Western/IP lysis buffer on ice. The beads were mixed with SDT lysis buffer (4% SDS, 100 mM DTT, 100 mM Tris HCl) for elution. Co-IP samples were analyzed by western blot assay.

### Pull-down assay

Purified 6×His tag peptide or His-SYTL4 protein was incubated with tubulins and 50 µl of nickel-nitrilotriacetic acid (Ni^2+^-NTA) beads (Thermo Scientific, USA) in 500 µl of western/IP lysis buffer (Beyotime) at 4 °C overnight. Subsequently, the His pull-down products were washed three times. Laemmli buffer was used to elute the protein, which was further detected by western blot with anti-α-tubulin and anti-SYTL4 antibodies.

### Orthotopic nude mouse models and treatment

To establish orthotopic models, MDA-MB-231 cells expressing shNC or shSYTL4 (5 × 10^6^ cells) were resuspended in 50 µL of PBS and 50 µL of Matrigel (BD Biosciences, USA) and injected directly into mammary fat pads of 4-6-week-old female BALB/c nude mice. When the average tumor volumes were 50-100 mm^3^ after implantation, the mice bearing each cell line were randomly assigned to two groups, namely, the vehicle or paclitaxel group. Vehicle or paclitaxel (10 mg/kg) was administered by intraperitoneal injection every 2 days for 9 times. Three days after the last dose, the mice were sacrificed. Tumor volumes were calculated as V = L × W × W / 2, where L is the length (longest dimension) and W is the width (shortest dimension). A paired t test was performed to compare the tumor volumes between groups. The animal study protocol was approved by the Animal Ethics Committee of the Fudan University.

### Immunohistochemistry staining

Immunohistochemistry (IHC) was performed and evaluated using a two-step method as described previously 39. Immunostaining was performed on the TNBC cohort C using tissue microarrays (TMAs). A rabbit polyclonal antibody against SYTL4 (Abcam, USA.) was applied to the TMAs. IHC staining of SYTL4 was mainly found in the cytoplasm of tumor cells. For the quantification of SYTL4 expression, both the staining intensity and the percentage of stained cells were evaluated as the histological score (H-score) 39. For statistical analysis, H-scores (ranging from 0-12) of 0 to 7 were considered low expression and scores of 8 to 12 considered high expression.

### Statistical analysis

All numerical data are expressed as the mean ± SD. The data were analyzed using a two-sided Student's *t*-test or a one-way ANOVA test. Comparisons of tumor characteristics were performed using the χ^2^ test. Survival curves were constructed using the Kaplan-Meier method, and the univariate survival difference was determined with the log-rank test. Unadjusted hazard ratios with 95% CIs were calculated using Cox proportional hazards models. All statistical analyses were performed using GraphPad Prism, version 7.0 (GraphPad Software, USA) or Stata statistical software, version 14.1 (StataCorp, USA). A two-sided *P* < 0.05 was considered significant. * *P* < 0.05; ** *P* < 0.01; *** *P* < 0.001.

Further details are shown in the supplementary material.

## Results

### Identification of chemoresistance-related genes in TNBC under neoadjuvant chemotherapy

**Figure 1:**
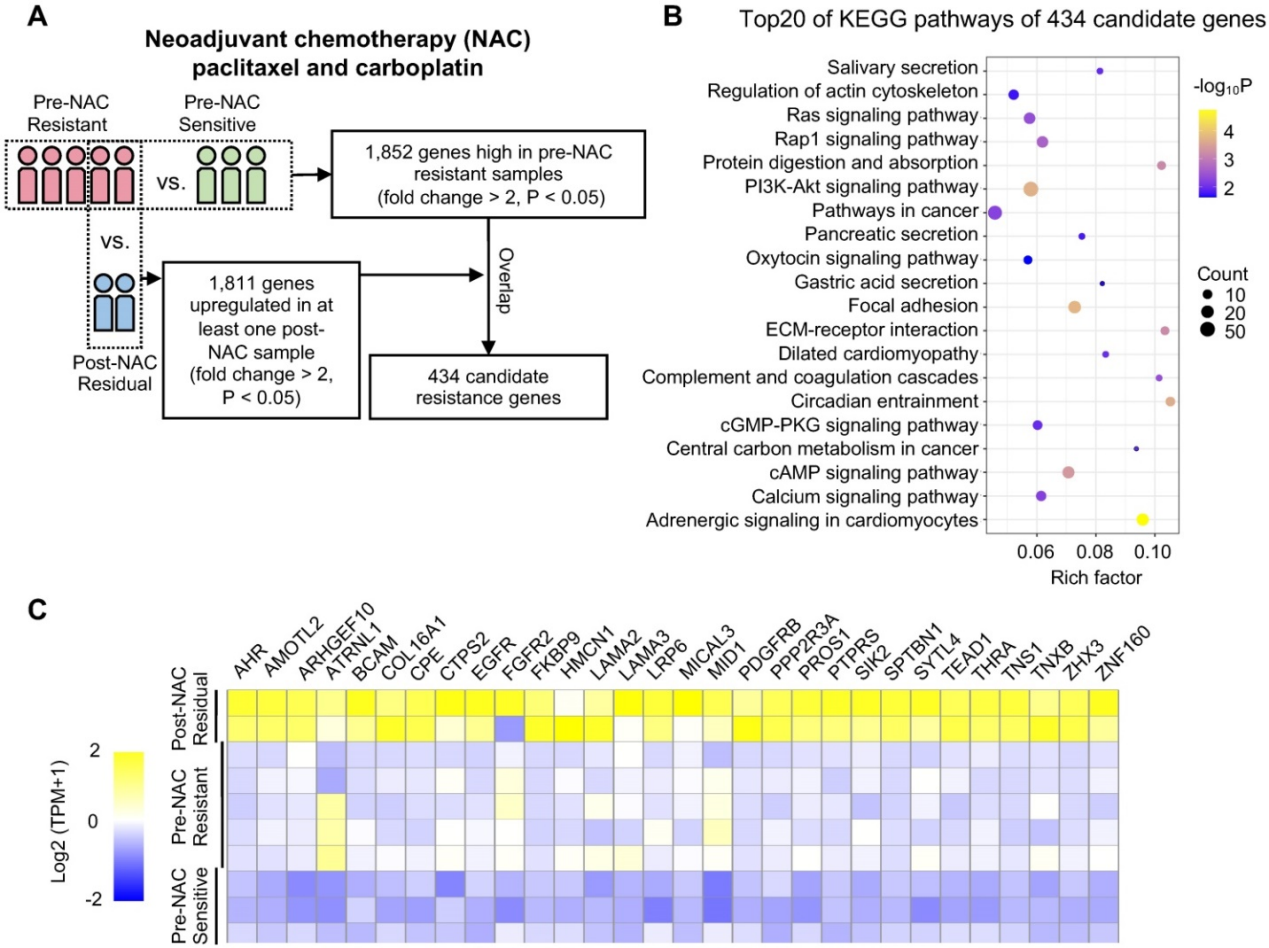
** Procedure for selecting differentially expressed genes associated with chemoresistance.** (**A**) Schematic diagram of overlapping candidate genes from RNA-seq data of triple-negative breast cancer (TNBC) samples that underwent neoadjuvant chemotherapy (NAC). (**B**) Top 20 of Kyoto Encyclopedia of Genes and Genomes (KEGG) pathways of 434 candidate genes as shown in (A). (**C**) Heatmap of the 30 top-ranked genes in 434 candidates above. The ranking standard was described in the methods.

First, 8 patients with TNBC who underwent neoadjuvant paclitaxel and carboplatin regimen were divided into resistant (n = 5) and sensitive (n = 3) groups according to their response (Table S1). We next sequenced 10 tumor samples obtained from these eight patients by RNA-seq (Figure 1A). These 10 TNBC samples were classified into the following groups: pre-NAC resistant (n = 5); pre-NAC sensitive (n = 3); and post-NAC residual (n = 2). Compared with pre-NAC sensitive samples, 1,852 genes were upregulated in the pre-NAC resistant samples, of which enriched pathways are shown in Figure S1A-B. In all, 1,253 genes were highly expressed in both post-NAC residuals compared with pre-NAC resistant samples, and 18,11 genes were high in at least one patient (Table S2). Enriched pathways of these 1,253 genes are shown in Figure S1C-D. Common signatures between post-NAC residual versus pre-NAC resistant and pre-NAC resistant versus pre-NAC sensitive included epithelial mesenchymal transition (EMT), myogenesis, and angiogenesis. With regard to the KEGG collection, common signatures included focal adhesion, calcium signaling pathway, cAMP signaling pathway, ECM-receptor interaction, the PI3K-Akt signaling pathway, and the Ras signaling pathway. To narrow down the gene numbers in the list for the next screening assay, we overlapped these genes and idendified 434 common genes upregulated in both comparisons; these genes were enriched in ECM-receptor interactions, focal adhesion, PI3K-Akt signaling, and the regulation of actin cytoskeleton pathways (Figure 1B). These 434 genes may be predictive of response to paclitaxel and carboplatin combination therapy.

Because we only sequenced 10 NAC samples, we next applied a list of scoring standards to rank candidate genes (Supplementary Methods). One key standard is that the gene must be predictive of pCR in at least one public taxane-based NAC cohort. Thirty top genes were selected for further functional exploration (Figure 1C), including SYTL4, BCAM, CTPS2, FKBP9, LAMA3, PDGFRB, and ZNF160 (Table S3).

### *In vitro* siRNA pool functional screen identified SYTL4 as a chemoresistant gene

**Figure 2:**
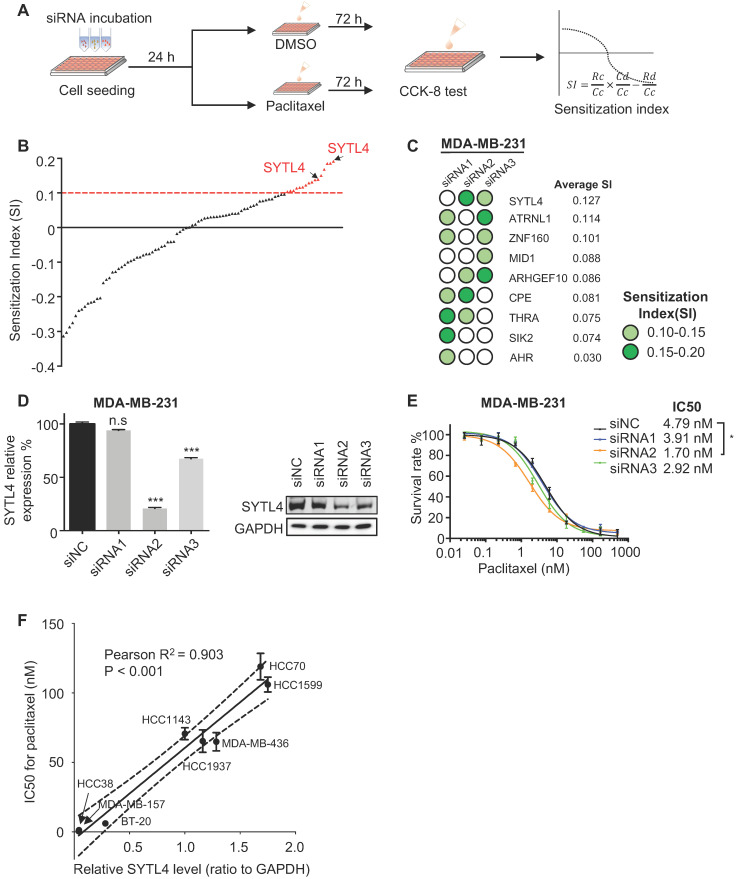
** Functional screening based on an siRNA pool assay in MDA-MB-231 cells.** (**A**) Schematic diagram of the siRNA-based screening. Sensitization index (SI) was described in the Methods section. Higher SI indicates a higher synergistic effect of siRNA with paclitaxel. (**B**) Sensitization index (SI) distribution curve. Dots in red: siRNA targets with SI > 0.1. (**C**) Average SI of three targets of genes from (B). (**D**) Relative gene expression of SYTL4 in MDA-MB-231 cells after siSYTL4 treatment. Left: qRT-PCR quantification of mRNA level (mean ± SD, n = 3). 2^-∆∆Ct^ was used and GAPDH was set as the inner control. Right: western blot analysis of SYTL4 expression. (**E**) IC50 of paclitaxel in MDA-MB-231 cells with siSYTL4 knockdown. Two-way ANOVA test was used to compare the effect of siRNA2 to siNC. (**F**) Correlation between relative SYTL4 protein level and IC50 of paclitaxel in TNBC cells. Pearson's correlation *R^2^* was calculated and tested. SYTL4 protein level was estimated by quantification of the gray intensity of western blot bands as shown in Figure S2C. * *P* < 0.05; ** *P* < 0.01; *** *P* < 0.001; n.s: not significant.

Next, we performed an siRNA pool-based screening of these above 30 genes. MDA-MB-231 cells (representing TNBC) were seeded, transfected, treated and measured using the CCK-8 assay (Figure 2A). The degree of paclitaxel sensitization calculated by SI equation across all targets distributed in a reverse S-shaped curve (Figure 2B). The top genes were ranked by the average SI (Figure 2C). Some reported chemoresistant genes, such as AHR 40 and SIK2 41, were present as well, confirming the reliability of our screening method. SYTL4 had the highest average SI. The knockdown efficiency of siRNAs targeting SYTL4 in MDA-MB-231 cells was validated by qRT-PCR and western blot (Figure 2D). The relative SYTL4 expression was down to approximately 20% of baseline by siRNA2, with IC50 of paclitaxel being decreased by 2.8-fold (Figure 2E). Utilizing public data, we found a significant positive correlation between SYTL4 mRNA expression level and the IC50 value for paclitaxel (Pearson's *R^2^* = 0.71, *P* = 0.004; Figure S2A) but not for vinorelbine (Pearson's *R^2^*=0.33, *P*=0.108; Figure S2B). The SYTL4 protein level was quantified by western blotting in another eight TNBC cell lines (Figure S2C). Simultaneously, the paclitaxel sensitivity of each cell line was assessed. A positive correlation between SYTL4 expression and IC50 value of paclitaxel was observed (Pearson's *R^2^* = 0.90, *P* < 0.001; Figure 2F). Given that SYTL4 was identified from patients treated with both paclitaxel and carboplatin, we also assessed the role of SYTL4 in carboplatin sensitivity. As shown in Figure S2D, knocking down SYTL4 did not alter the carboplatin sensitivity of MDA-MB-231 cells. The synergy scores of carboplatin combined with paclitaxel were calculated by SynergyFinder 2.0 42. Knocking down SYTL4 did not alter the additive interaction between these two drugs in MDA-MB-231 cells (Figure S2E). Of note, silencing SYTL4 did not alter the sensitivity of MDA-MB-231 cells to other nontaxane drugs-vinorelbine (a microtubule-destabilizing agent) or doxorubicin (a DNA-damaging agent) (Figure S2F). We thus chose SYTL4 as the leading potential candidate for paclitaxel resistance in further exploration.

### The prognostic value and clinical relevance of SYTL4 in TNBC

**Figure 3:**
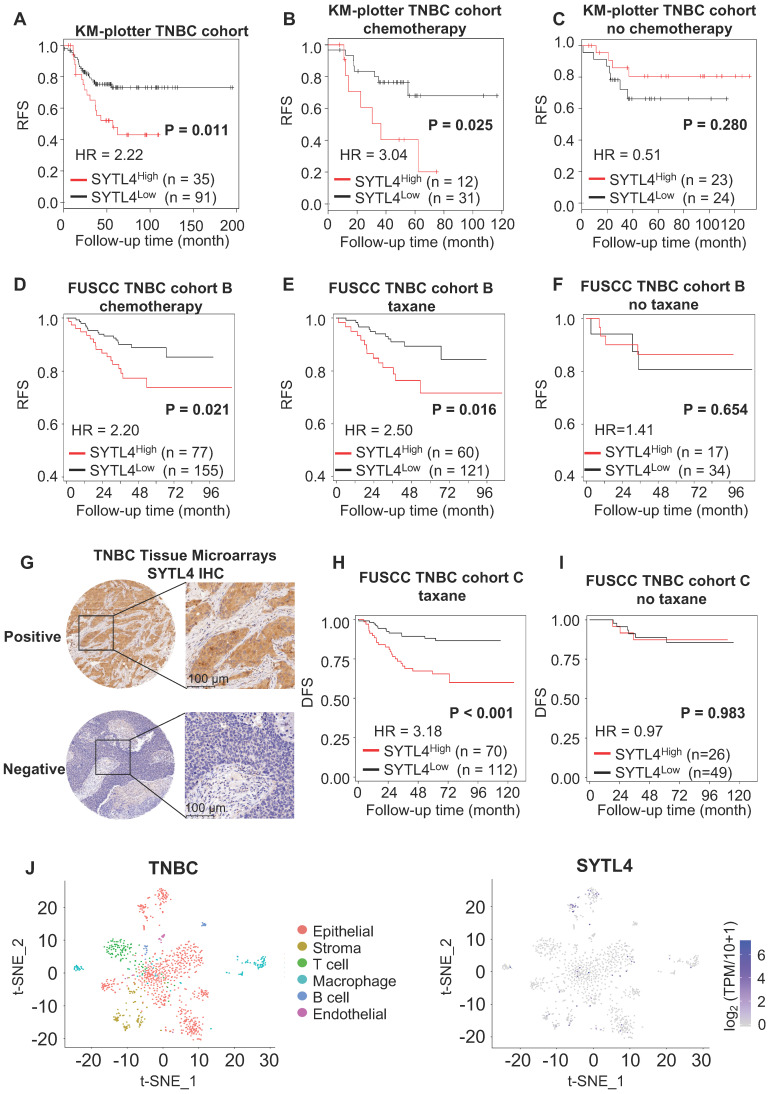
** SYTL4 correlated with poor prognosis in taxane-treated TNBC cohorts.** (**A-C**) Survival analysis of SYTL4 expression in TNBC cohort from KM-plotter 43. (**D-F**) Survival analysis of SYTL4 expression in FUSCC TNBC cohort B (n = 232). (**G**) Representative images of SYTL4 expression in tumor tissue microarrays of TNBC by IHC. (**H, I**) Survival analysis of SYTL4 protein level and in FUSCC TNBC cohort C (n = 182). SYTL4 protein level was estimated by IHC as described in the Methods section. (**J**) t-SNE plot of all 1,069 classified TNBC cells, demonstrating separation of cells by cell type (left panel). Expression level and distribution of SYTL4 vary across cells (right panel). The hazard ratio (HR) was calculated by univariate Cox regression. DFS, disease-free survival; FUSCC, Fudan University Shanghai Cancer Center; IHC, immunohistochemistry; RFS, recurrence-free survival; TNBC, triple-negative breast cancer; t-SNE, t-distributed stochastic neighbor embedding.

We next assessed the prognostic value of SYTL4 in TNBC. In a public TNBC cohort from KM-plotter 43, high SYTL4 expression showed worse recurrence-free survival (RFS) in the full TNBC set (hazard ratio [HR] = 2.22, *P* = 0.011; Figure 3A). The HR of SYTL4 expression increased to 3.04 in chemotherapy-treated TNBC (*P* = 0.025; Figure 3B). In contrast, the prognostic value of SYTL4 mRNA was not significant in the subset without chemotherapy (Figure 3C). Additionally, in our FUSCC TNBC cohort B 5, a high level of SYTL4 mRNA indicated worse RFS in 232 TNBC cases who received adjuvant chemotherapy (HR = 2.20, *P* = 0.043; Figure 3D), specifically in taxane-treated patients (HR = 2.50, *P* = 0.027; Figure 3E) but not in those without taxane treatment (Figure 3F). Tumors with high levels of SYTL4 were enriched in MES subtypes (*P* = 0.001; Table 1) and enriched in myogenesis, EMT, apical junction and angiogenesis signatures by GSEA (Table S4). The protein level of SYTL4 in TNBC was evaluated by IHC on TMAs in another TNBC cohort (Figure 3G). High protein level of SYTL4 significantly correlated with poor disease-free survival (DFS) in taxane-treated patients (HR = 3.18, *P* < 0.001; Figure 3H), but not in those without taxane treatment (*P* = 0.983; Figure 3I). These results indicated SYTL4 as a poor prognostic indicator for taxane-treated TNBCs.

We next compared the mRNA expression of SYTL4 between 1) pre-NAC samples from pCR (n = 12) and non-pCR patients (n = 12) and 2) pre- and post- NAC samples from non-pCR patients (n = 12) in a TNBC cohort that underwent taxane-containing NAC. TNBC patients who did not reach a pCR had a significantly higher level of SYTL4 expression (*P* < 0.001; Figure S3A). After NAC, the SYTL4 mRNA level increased at 1.2- to 3-fold after NAC in approximately 75% of non-pCR patients (*P* < 0.01; Figure S3B). Within public breast cancer cohorts that underwent NAC, SYTL4 was also highly expressed in non-pCR patients (GSE22513) and upregulated in post-NAC samples (GSE32603) (Figure S3C-D). These results validated the reliability of SYTL4 in NAC samples.

Since single-cell sequencing helps identify intratumor heterogeneity of breast cancer, we explored the distribution of SYTL4 expression utilizing single-cell data of TNBC 44. SYTL4 was highly expressed in a cluster of epithelial tumor cells (Figure 3J), which were enriched in metabolic-related pathways (Table S5).

In summary, these data suggested that highly expressed SYTL4 indicated poor prognosis and chemotherapy response in TNBC patients.

### SYTL4 promoted paclitaxel resistance in TNBC *in vitro* and *in vivo*

To explore the phenotype of SYTL4-mediated resistance* in vitro* and* in vivo*, we first evaluated SYTL4 expression across breast cancer cell lines in publicly available data (GSE58135). SYTL4 was highly expressed in most ER-positive and HER2-positive cells (Figure S4A). Protein SYTL4 expression was similar to its mRNA expression pattern (Figure S4B). In tumors, the SYTL4 mRNA level was significantly lower in the basal-like subtype than luminal-like ones within The Cancer Genome Atlas (TCGA) cohort. However, within the FUSCC TNBC cohort, SYTL4 was expressed highly in the MES subtype, a subtype possessing the poorest survival 5 (Figure S4C), suggesting a distinct role of this protein in TNBCs.

**Figure 4:**
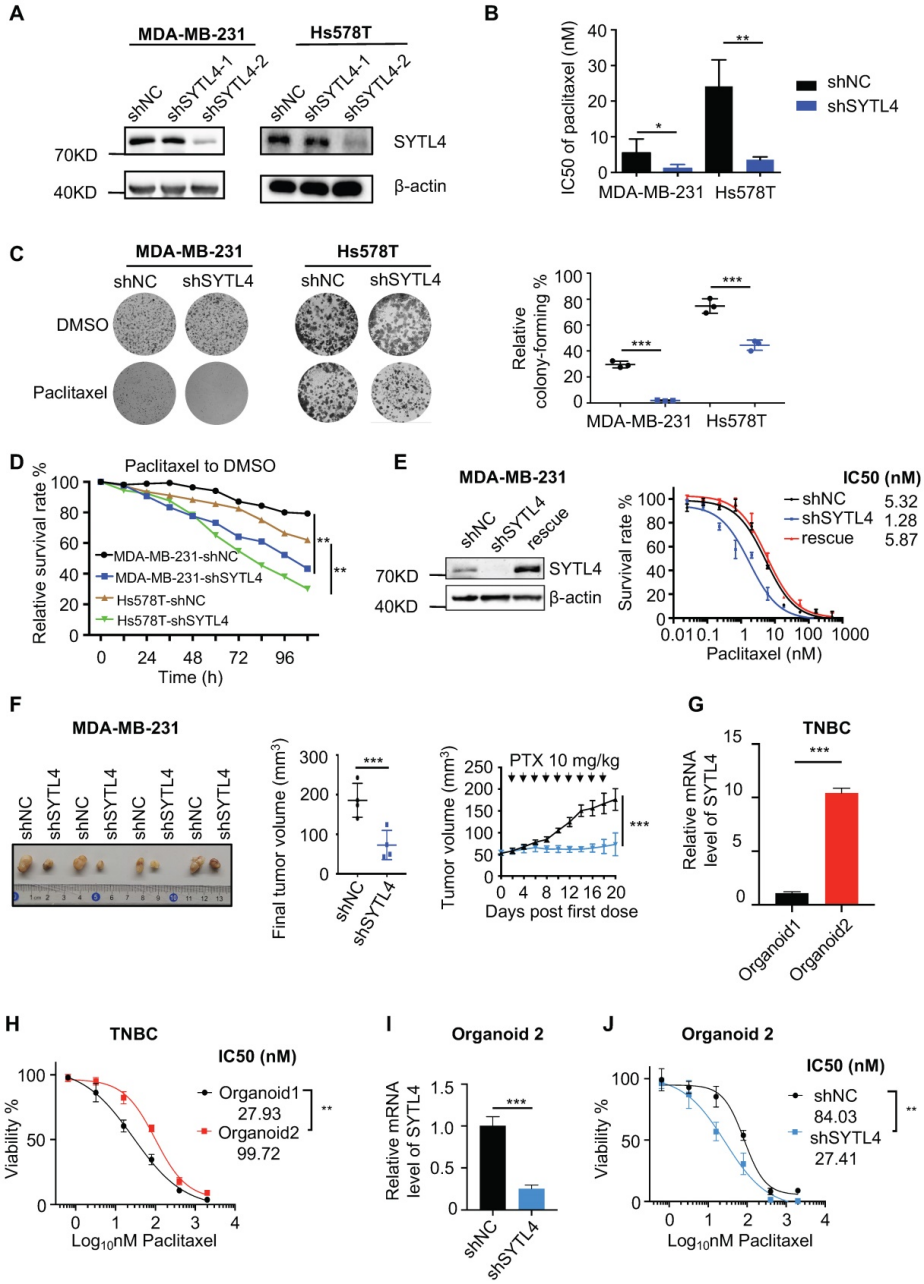
** Knocking down SYTL4 increased the paclitaxel sensitivity in TNBC.** (**A**) Western blot analysis of SYTL4 expression in MDA-MB-231 and Hs578T cells. The two short hairpin RNA (shRNA) target sequences were described in the Methods section. (**B**) IC50 of paclitaxel in cells with SYTL4 knockdown tested by the CCK-8 assay. The data represents three independent assays (mean ± SD, n = 3). (**C**) SYTL4 knockdown inhibited the cell colony-formation of MDA-MB-231 and Hs578T cells under paclitaxel treatment (1 nM). The relative survival rate was calculated by dividing colony numbers under paclitaxel treatment into colony numbers under DMSO. The data represent three independent assays (right) (mean ± SD, n = 3). (**D**) IncuCyte-based real-time imaging analysis of cell growth with the treatment of paclitaxel. The relative cell survival rate on the Y axis was calculated by dividing the cell numbers under paclitaxel treatment by the cell numbers under DMSO treatment. (**E**) Rescuing SYTL4 expression increased the IC50 of paclitaxel in MDA-MB-231. Western blot analysis of SYTL4 expression (left). IC50 of paclitaxel in MDA-MB-231 (right). (**F**) SYTL4 knockdown inhibited tumor growth in nude mice after sequential paclitaxel (PTX) treatment. MDA-MB-231 cells with shNC or shSYTL4 were transplanted into nude mouse mammary fat pads in pairs as described in the Methods section. Arrows represent paclitaxel (10 mg/kg) treatment in tumor-bearing mice. Final tumor images were shown (left). Final tumor volume was calculated (middle) (mean ± SD, n = 4) and tested by paired t test. *In vivo* growth curves quantified by tumor volume were illustrated (right) and tested by two-way ANOVA test. (**G**) qRT-PCR analysis of the relative mRNA levels of two TNBC organoids and tested by unpaired t test (mean ± SD, n = 3). Human 18S rRNA was chosen as the reference gene. (**H**) Dose-response curves of organoid 1 (SYTL4 low expression) and organoid 2 (SYTL4 high expression) (mean ± SD, n = 3, two-way ANOVA test). (**I**) qRT-PCR analysis of the relative mRNA levels of TNBC organoid 2 with shNC or shSYTL4 and tested by unpaired t test (mean ± SD, n = 3). (**J**) Dose-response curves of organoid 2 (shNC vs. shSYTL4) (mean ± SD, n = 3, two-way ANOVA test). * *P* < 0.05; ** *P* < 0.01; *** *P* < 0.001; n.s: not significant.

We stably silenced SYTL4 expression through short hairpin RNA (shRNA) in MDA-MB-231 and Hs578T cells, two TNBC cell lines. Western blot analysis showed that shRNA target 2 worked well in both cell lines (Figure 4A).

As expected, the IC50 value in shSYTL4-2 cells (hereafter referred to as shSYTL4) was approximately 5-fold lower in MDA-MB-231 cells and 7-fold lower in Hs578T cells compared with shNC cells (Figure 4B). We also performed IC50 assays in MDA-MB-231 cells utilizing another two working siRNAs targeting SYTL4 (Figure S5A). Knocking down SYTL4 decreased IC50 values significantly (Figure S5B). Consistent with this finding, colony-formation assays (Figure 4C) and proliferation assays (Figure 4D) showed that SYTL4 knockdown significantly improved sensitivity to paclitaxel without affecting cell proliferation in both cell lines. Rescuing SYTL4 expression in MDA-MB-231-shSYTL4 cells improved the IC50 value to an equivalent level in MDA-MB-231-shNC cells (Figure 4E). In addition, overexpressing SYTL4 increased the resistance of MDA-MB-231 and Hs578T cells more resistant to paclitaxel (Figure S5C-H). Furthermore, silencing SYTL4 enhanced sensitivity to paclitaxel in the BALB/c nude mouse model (Figure 4F) without affecting tumor growth (Figure S6A-B). These data indicated that altering the SYTL4 level could affect the sensitivity of TNBC cells to paclitaxel *in vitro* and *in vivo*.

Patient-derived organoids (PDO) is a reliable model reflecting the chemosensitivity of individual patients 34. Hoechst/PI staining showed that treating PDO with paclitaxel induced evident apoptosis (Figure S6C). SYTL4 high-expression TNBC organoids were more resistant to paclitaxel (Figure 4G-H). Meanwhile, knocking down SYTL4 in organoids improved the tumor sensitivity to paclitaxel (Figure 4I-J), suggesting that SYTL4 may serve as a potential therapeutic target for paclitaxel resistance in TNBC.

Altogether, *in vitro* and *in vivo* models validated that SYTL4 promoted paclitaxel resistance in TNBC.

### SYTL4 interacted with microtubules

**Figure 5:**
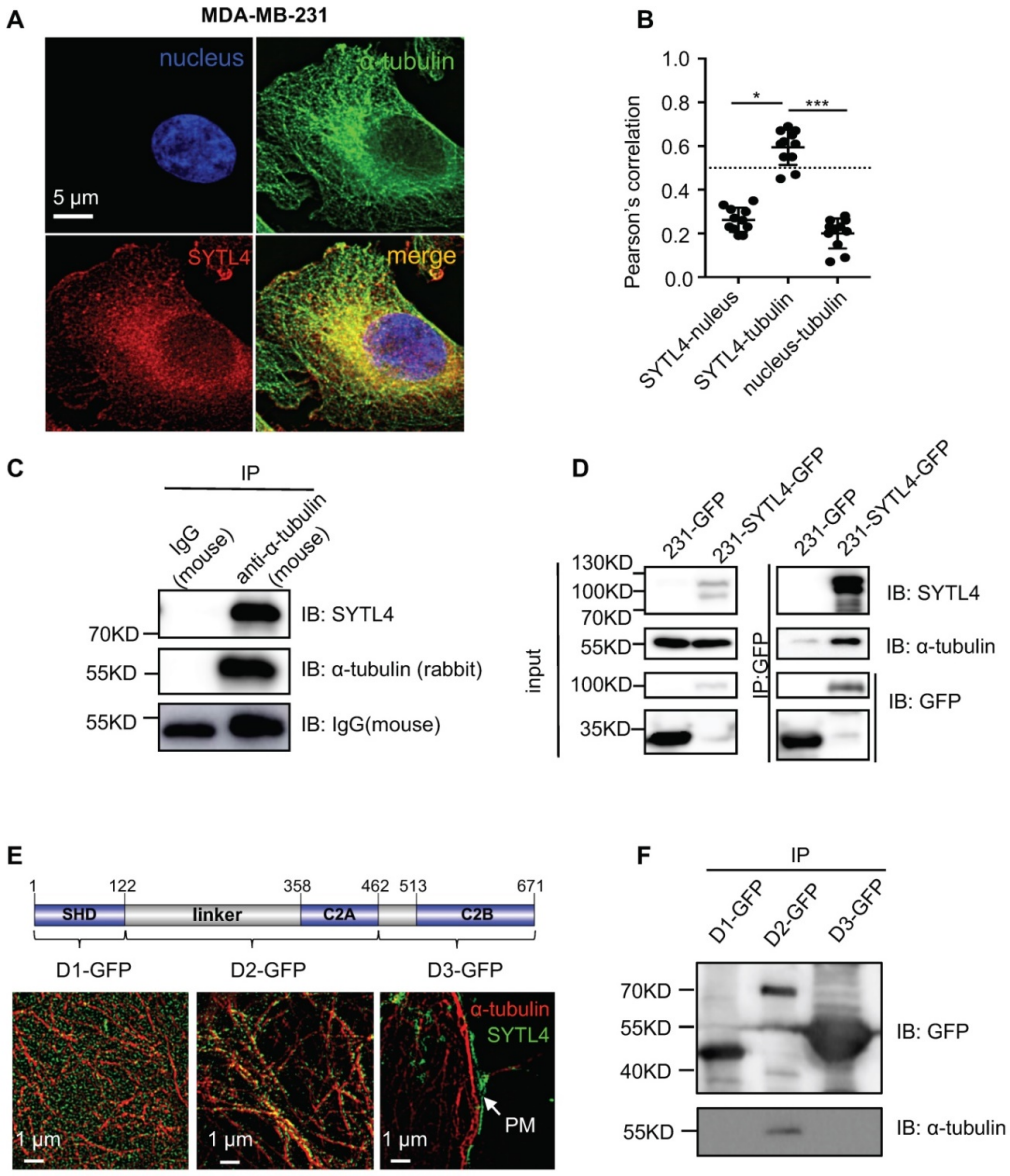
** SYTL4 colocalized and interacted with microtubules.** (**A**) SYTL4 colocalized with microtubules in MDA-MB-231 cells. Anti-SYTL4 and anti-α-tubulin antibodies were used and are described in the Methods section. Nuclei were stained by DAPI. Images were captured and deconvolved by DeltaVision microscopy. (**B**) Colocalization analysis by Pearson's correlation. The colocalization of SYTL4 and α-tubulin as shown in (A) was estimated by calculating the Pearson's correlation of their fluorescence intensities. The data represents the mean ± SD estimated in at least 10 cells. (**C**) Coimmunoprecipitation (Co-IP) analysis of SYTL4-overexpressing MDA-MB-231 cells using anti-α-tubulin antibody. (**D**) Co-IP analysis of SYTL4-GFP-overexpressing MDA-MB-231 cells using anti-GFP nanobody-coated agarose beads. (**E**) Colocalization analysis of D1, D2 and D3 with microtubule by structured illumination microscopy (SIM) 293T cells. PM: plasma membrane. (**F**) Co-IP analysis of the interaction between SYTL4 D1, D2, D3 and microtubule. Co-IP assay was performed in 293T cells using anti-GFP beads. * *P* < 0.05; ** *P* < 0.01; *** *P* < 0.001; n.s: not significant.

According to COMPARTMENTS Experimental Protein Localization Evidence Scores, SYTL4 is one of the 494 proteins localized to the microtubule cytoskeleton 45, 46. We thus visualized the distribution of SYTL4 by deconvolution-based fluorescence microscopy (Figure 5A). SYTL4 colocalized with α-tubulin, as indicated by the Pearson's correlation (Figure 5B). Of note, MDA-MB-231 cells with SYTL4 knockdown showed specific loss of staining by immunofluorescence (Figure S7A-B), confirming antibody specificity for SYTL4. Furthermore, live-cell imaging in MDA-MB-231 cells showed the colocalization between SYTL4-RFP and microtubules (Figure S8A-B). In addition, the SYTL4-tubulin complex could be coimmunoprecipitated (co-IP) in cell lysates (Figure 5C-D), suggesting their interaction. Finally, we questioned which domain of SYTL4 was responsible for this interaction. Three parts of SYTL4, the SHD domain (D1), the linker-C2A domain (D2) and the C2B domain (D3), were overexpressed in 293T cells individually as D1-GFP, D2-GFP, and D3-GFP (Figure 5E). Super-resolution imaging analysis through structured illumination microscopy (SIM) suggested that D2 attached to the surface of microtubules, while D1 exhibited a freely distributed pattern and D3 specifically localized near the plasma membrane (PM) (Figure 5E). Consistent with this finding, co-IP assay confirmed that D2 was responsible for interacting with microtubules (Figure 5F). These data indicated that SYTL4 interacted with microtubules through the region containing the linker-C2A domain.

### SYTL4 induces microtubule instability in TNBC cells

**Figure 6:**
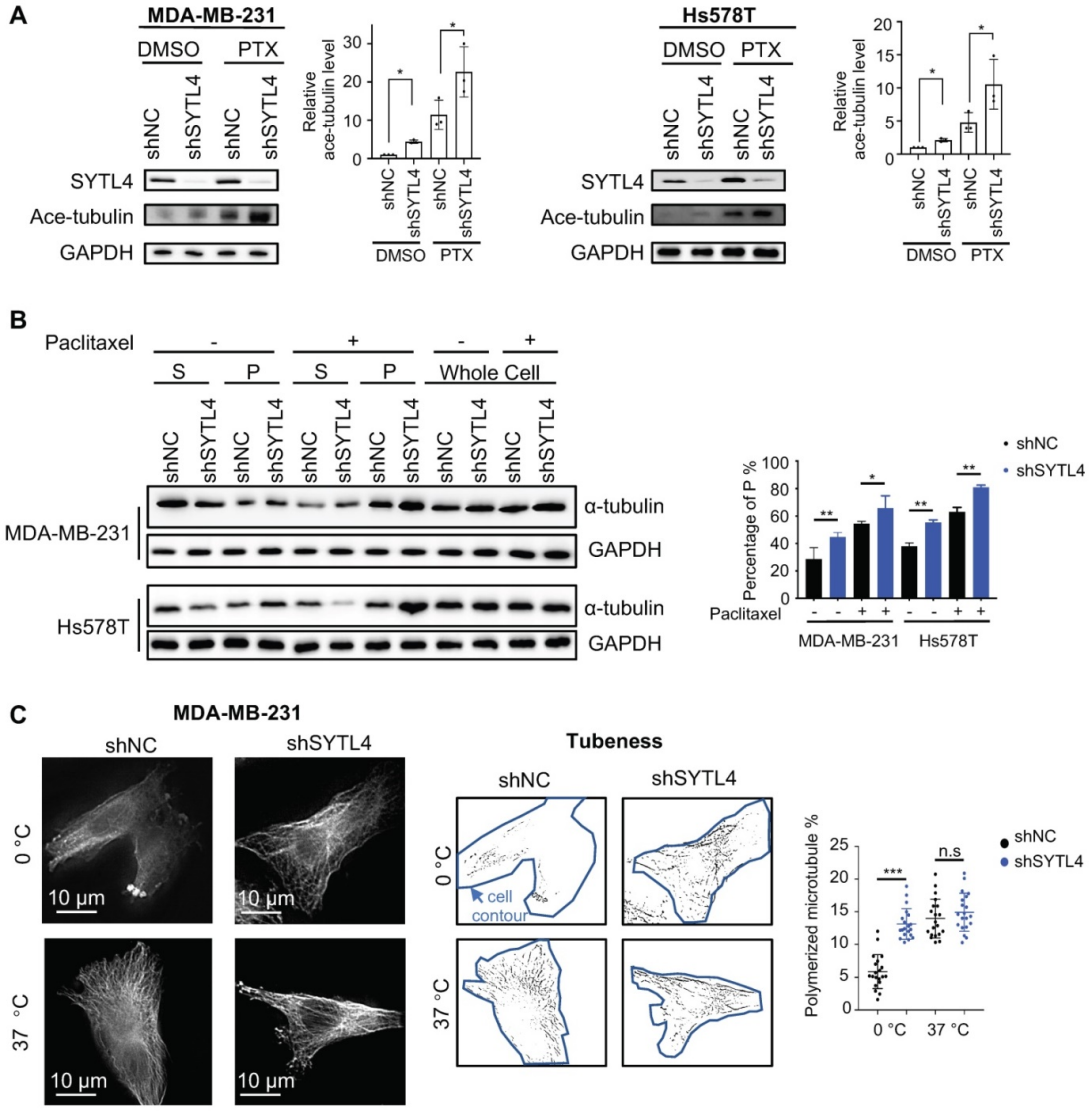
** Knocking down SYTL4 enhanced microtubule stability in TNBC.** (**A**) Western blot analysis of microtubule acetylation in MDA-MB-231 and Hs578T cells with or without PTX (paclitaxel) treatment. Band intensity was estimated by Fiji. The data represent the band intensity of ace-tubulin relative to the baseline intensity level of shNC cells under DMSO treatment (mean ± SD, n = 3, one-way ANOVA test). GAPDH was used for normalization. (**B**) Western blot analysis of microtubule stability in MDA-MB-231 and Hs578T cells (left panel). The lysates were separated into pellet fractions (*P*) containing microtubules and supernatant fractions (*S*) containing soluble tubulin. The band intensity was estimated by Fiji. The percentage of assembled tubulin was calculated as follows: 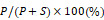. The data represent the mean ± SD of three independent assays (one-way ANOVA test, right panel). (**C**) Immunofluorescence analysis of microtubule stability in MDA-MB-231 cells at 0 °C and 37 °C (left). Tubule-like structures were recognized by Fiji using the Tubeness plugin (middle) as described in methods. The percentage (%) of polymerized microtubules was calculated by dividing the area of tubule-like structure into the region inside the cell contour (right) (mean ± SD, n = 20, one-way ANOVA test). * *P* < 0.05; ** *P* < 0.01; *** *P* < 0.001; n.s: not significant.

Paclitaxel-induced cytotoxicity depends primarily on stabilizing microtubules 47. Enhanced paclitaxel sensitivity could arise from enhanced microtubule stability. Because SYTL4 interacted with microtubules, we questioned whether SYTL4 confers paclitaxel resistance by affecting microtubule stability. The state of tubulin acetylation (ace-tubulin), a marker for stable microtubules 48, was detected by western blot. The data showed that SYTL4 knockdown increased the acetylation levels of tubulin in both MDA-MB-231 and Hs578T cells with or without paclitaxel treatment (Figure 6A). In addition, overexpressing SYTL4 decreased the level of acetylated tubulin to 50% in MDA-MB-231 and Hs578T cells (Figure S8C). Percentages of intracellular polymerized tubulin in both MDA-MB-231 and Hs578T cells were quantified. We found that stable microtubule (P) was significantly increased after knocking down SYTL4 with or without paclitaxel treatment (Figure 6B). Furthermore, SYTL4 knockdown also stabilized microtubules against cold treatment, a microtubule depolymerization inducer (Figure 6C). Another two siRNAs targeting SYTL4 (siRNA4 and siRNA5) validated this phenotype (Figure S9A-B).

**Figure 7:**
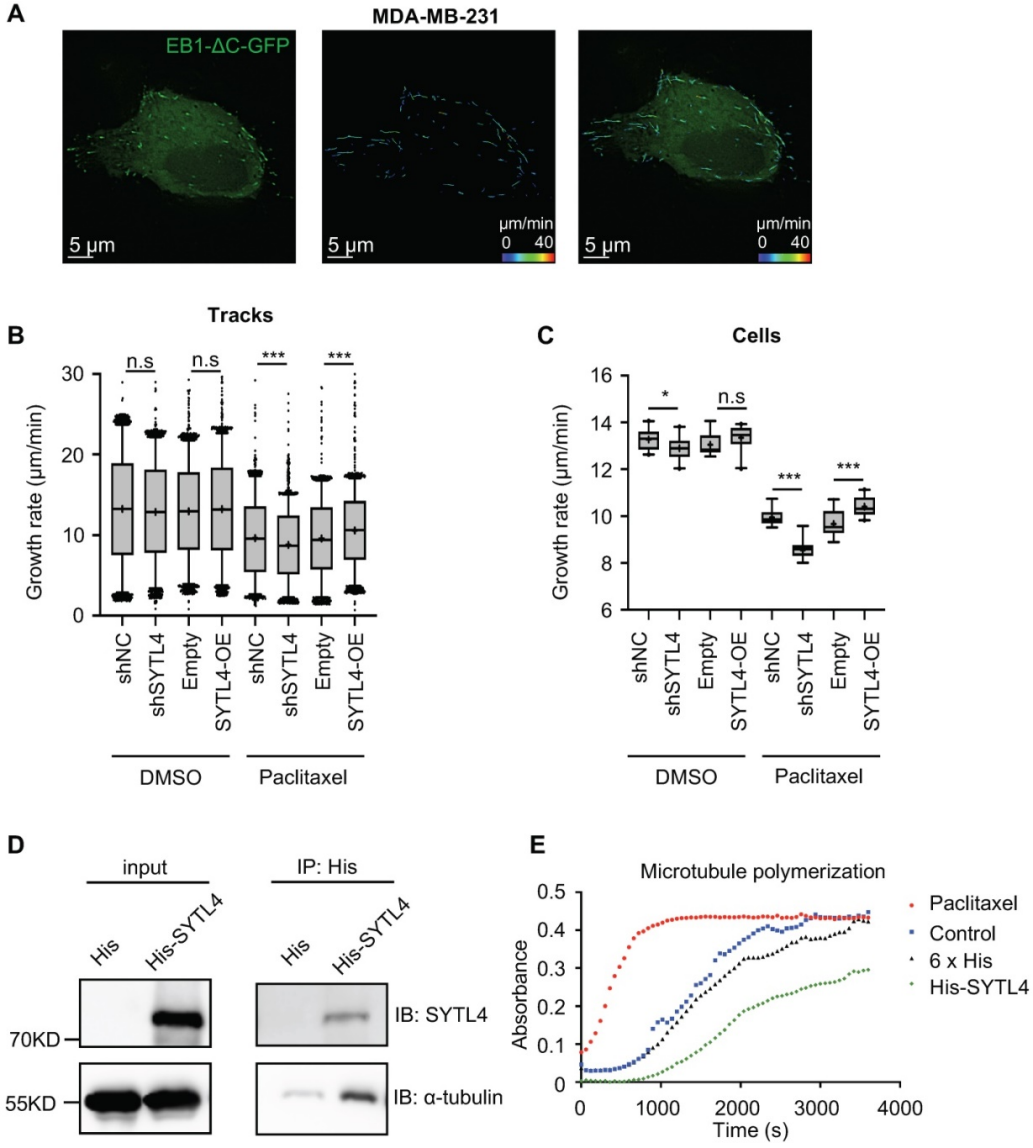
** SYTL4 increased microtubule dynamics via directly destabilizing microtubule polymers in TNBC.** (**A**) Representative image of EB1 comets and track overlays in an MDA-MB-231 cell. This image is a snapshot from a 30 s time-lapse recording (scale bar, 5 µm). Spectrum lines represented overall EB1-ΔC-GFP comet movement for a 30-s time-lapse recording. Time-lapse images were acquired every 2 s for 30 s. See the Methods for a more thorough explanation. (**B**) Growth rates of tracks with or without paclitaxel treatment in MDA-MB-231 cells. Microtubule growth rates were calculated by directly observing the EB1-ΔC-GFP comets. Box plots indicate the 5th percentile (bottom boundary), median (middle line), 95th percentile (top boundary) and mean value (+). Points represent outliers. One-way ANOVA test. (**C**) Growth rates per cell means with or without paclitaxel treatment in MDA-MB-231 cells. Box plots indicate the 5th percentile (bottom boundary), median (middle line), 95th percentile (top boundary) and mean value (+) (n = 20 cells per condition). One-way ANOVA test. (**D**) SYTL4 directly interacted with α-tubulin. A pull-down assay was performed in a mixture of purified His-SYTL4 protein and tubulin as described in the Methods section. (**E**) SYTL4 inhibited *in vitro* microtubule polymerization. Paclitaxel was used as a positive control, and 6x His was used as a negative control. His-tagged SYTL4 was added to the microtubule polymerization solution.

EB1-ΔC-GFP, the fluorescently labeled microtubule plus-end tracking protein, has become a practical tool for monitoring microtubule dynamics in live cells 38. EB1-ΔC binds to the plus ends of microtubule as a “comet,” which enables the quantification of microtubule growth rate. To assess the effects of SYTL4 on microtubule dynamics, we monitored over 15,000 EB1 tracks in control, SYTL4-knockdown and SYTL4-overexpressing MDA-MB-231 cells (Figure 7A). We observed a trend, though not a significant one, for decreased the growth rates of tracks after knocking down SYTL4 (P = 0.051, Figure 7B). Growth rates of tracks after paclitaxel treatment further revealed that SYTL4 knockdown significantly decreased the microtubule growth rate of all tracks (8.78 ± 0.12 μm/min vs. 9.64 ± 0.12 μm/min; Figure 7B) and per cell means (Figure 7C). After SYTL4 overexpression, we observed a significant increase in the growth rate of all tracks (10.59 ± 0.09 μm/min vs. 9.59 ± 0.10 μm/min; Figure 7B) and the per cell means (Figure 7C). Taken together, these data indicated that SYTL4 increased dynamic instability during microtubule growth.

**Figure 8:**
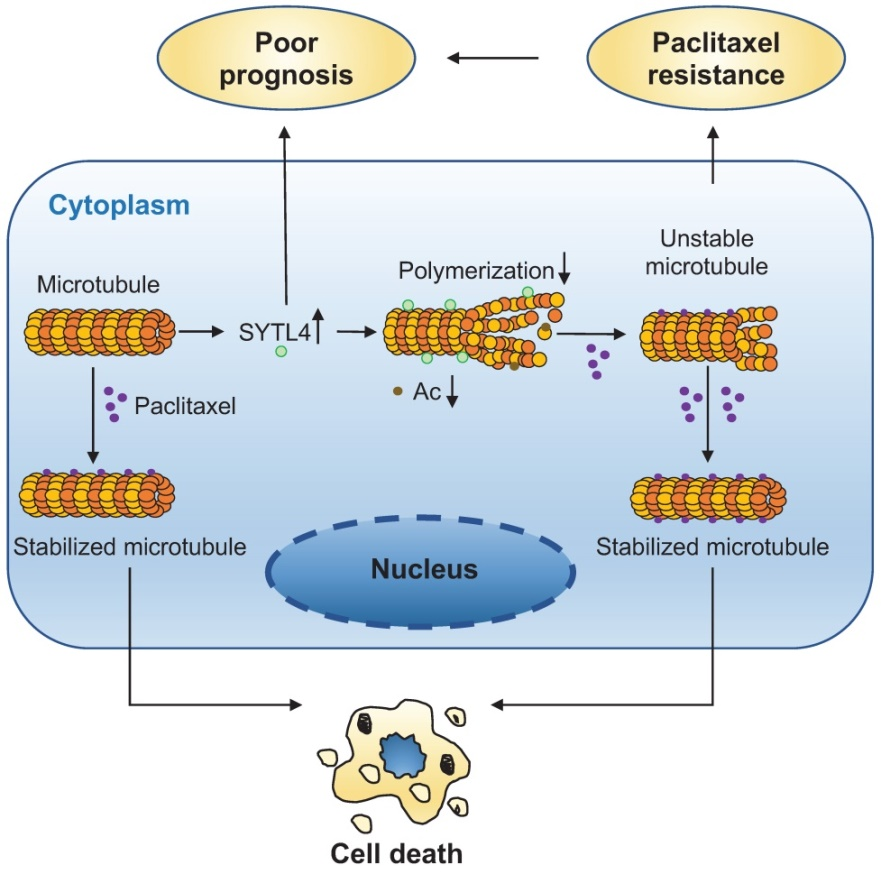
** A schematic diagram for explaining the role of SYTL4 in conferring paclitaxel resistance in TNBC.** Upregulated SYTL4 expression is correlated with poor prognosis in triple-negative breast cancer (TNBC). SYTL4 directly interacts with microtubules and inhibits the microtubule polymerization, thus increasing microtubule instability. Accordingly, the acetylation level (Ac) of microtubules decreases. Unstable microtubules require higher paclitaxel concentrations to keep stabilized and induce cell death, thereby mediating TNBC paclitaxel resistance.

The direct interaction between α-tubulin and SYTL4 was further validated by an *in-vitro* pull-down assay (Figure 7D). To determine whether SYTL4 could counteract microtubule polymerization, we conducted an *in vitro* microtubule polymerization assay. As a positive control, paclitaxel enhanced microtubule polymerization. Compared to the negative control, SYTL4 inhibited *in vitro* microtubule polymerization (Figure 7E). Taken together, these results demonstrated that SYTL4 increased the dynamic instability of microtubule polymers and thus counteracted the microtubule polymerization effect induced by paclitaxel (Figure 8).

## Discussion

Resistance to paclitaxel is a major obstacle to the acquired successful treatment of TNBC. Tumor tissue during NAC offers a great source of material for the identification of molecular markers associated with potential chemoresistance-associated alterations. However, tumors after chemotherapy are often paucicellular and contaminated with nontumor cells. To overcome this limitation, we used post-NAC samples from patients with progressive disease, where tumors cells have outgrown nontumor cells. Previous molecular profiling of chemotherapy-resistant breast cancers has identified Ras-ERK pathway activation, degradation of ECM, AKT1 signaling via mTOR, hypoxia, EMT, and angiogenesis as activated pathways related to chemoresistance 15, 16, 18. Similarly, we also identified the PI3K-Akt signaling pathway, the Ras signaling pathway, EMT, and angiogenesis. Coupled with functional screening *in vitro*, we report here that SYTL4 may be useful as a biomarker of paclitaxel resistance.

We observed that SYTL4 expression was higher in post-NAC residual samples, suggesting that this upregulated expression arises from the *in vivo* drug selection of treatment-refractory subpopulations or drug stimulation in response to chemotherapy 18, 49. Interestingly, we found that a low concentration of paclitaxel treatment did not alter SYTL4 expression in TNBC cells (data not shown). Future investigation is needed to determine why SYTL4 is highly expressed in chemoresistant TNBC tumors.

Our data have several clinical implications. First, the level of SYTL4 correlated with poor prognosis and taxane response in TNBC, providing a new diagnostic marker for the administration of chemotherapy. Second, altering SYTL4 expression in MDA-MB-231 cells did not change its sensitivity to nontaxane drugs, including vinorelbine, a vinca alkaloid that interferes with microtubule assembly 50. Thus, nontaxane-based regimens may benefit the TNBC patients with high-level SYTL4 expression. Third, downregulating SYTL4 expression via siRNA/shRNA knockdown or other techniques may provide a novel strategy to combat paclitaxel resistance in TNBC patients.

We clearly demonstrated a function of SYTL4 in the resistance to paclitaxel in at least two types of TNBC cells. Silencing of SYTL4 renders TNBC cells sensitive to paclitaxel both *in vitro* and *in vivo*, especially in PDO models, providing a new strategy to enhance paclitaxel sensitivity. In addition, we revealed SYTL4 as a novel microtubule-binding protein that decreased microtubule stability by counteracting microtubule polymerization. Importantly, SYTL4 was both predictive of response to taxane-based neoadjuvant chemotherapy and prognostic in taxane-treated TNBCs, which may serve as a candidate marker for predicting taxane response in TNBC.

Microtubules are essential components for vesicle transport. In turn, several molecular motors and Rab effectors have been shown to modulate microtubule stability 51. Several microtubule-destabilizers have been reported to induce paclitaxel resistance by increasing the microtubule instability 31, 36, 52. As a Rab27 effector, SYTL4 was previously identified to localize on intracellular vesicles and dock secretory granules to the plasma membrane for secretion through interacting with Rab3, Rab8 and Rab27 family members by the N-terminal Slp homology domain (SHD) 53, 54. In addition, the linker region could interact with Munc18-1, syntaxin-1a, and Stx3, and the C-terminal C2AB domain has phospholipid interaction sites 55, 56. Previous studies have illustrated the interplay between SYTL4 and kinesin-1 in linking granules along the cytoskeleton 57, 58. Our study explored the distribution of SYTL4 proteins in cells and showed that a proportion of SYTL4 proteins colocalized with microtubules. We showed that SYTL4 could directly interact with microtubules through its middle region containing linker and C2A domain. This interaction results in microtubule instability and decreases sensitivity to paclitaxel in TNBC.

One of the limitations of this study is that the initial gene-list was generated from a small sample size for the RNA-seq. However, we performed complementary analyses utilizing multiple public cohorts as well as a functional screening to narrow down the gene list and further validated the reliability of SYTL4 in another NAC cohort with a larger sample size. In addition, high SYTL4 expression correlated with myogenesis, EMT, apical junction and angiogenesis, suggesting a possible role of SYTL4 in the tumor microenvironment, which needs further assessment. It would also be interesting to investigate why SYTL4 is highly expressed in TNBC tumors but not in other breast cancer subtypes.

In conclusion, we suggest SYTL4 as a robust prognostic marker in taxane-treated TNBC, which may help in the selection of proper therapy and thus improve patient outcomes. In addition, we identified SYTL4 as a new microtubule destabilizer, enabling a better understanding of the regulatory mechanisms of microtubule dynamics in cancer cells.

## Supplementary Material

Supplementary figures and tables.

## Figures and Tables

**Table 1:** Correlation between clinicopathologic characteristics and SYTL4 mRNA expression in FUSCC TNBC cohort B

	SYTL4 low, n = 155 (%)	SYTL4 high, n = 77 (%)	*P*-value
Median follow-up (IQR) (mo)	40.8 (34.1-59.6)		-
**Age**			
18-49	58 (37.4)	29 (37.7)	0.971
≥50	97 (62.6)	48 (62.3)	
**Menopause**			
Yes	102 (65.8)	45 (58.4)	0.541
No	51 (32.9)	31 (40.3)	
N/A	2 (1.3)	1 (1.3)	
**Grade**			
2	18 (11.6)	11 (14.3)	0.387
3	126 (81.3)	57 (74.0)	
N/A	11 (7.1)	9 (11.7)	
**T stage**			
T1	53 (34.2)	28 (36.4)	0.744
T2-3	102 (65.8)	49 (63.6)	
**LN status**			
Negative	94 (60.6)	46 (59.7)	0.765
Positive	61 (39.4)	31 (40.3)	
**Surgery**			
BCS	3 (1.9)	1 (1.3)	0.220
Mastectomy	152 (98.1)	76 (98.7)	
**Radiotherapy**			
No	109 (70.3)	51 (66.2)	0.526
Yes	46 (29.7)	26 (33.8)	
**FUSCC subtype**			
BLIS	64 (41.3)	21 (27.3)	0.001
IM	43 (27.7)	14 (18.2)	
LAR	33 (21.3)	21 (27.3)	
MES	15 (9.7)	21 (27.3)	
**Mutation subtype**			
Aging	27 (17.4)	11 (14.3)	0.911
HRD	36 (23.2)	18 (23.4)	
MMR	16 (10.3)	10 (13.0)	
Mixed	22 (14.2)	9 (11.7)	
N/A	54 (34.8)	29 (37.7)	
**sTIL group**			
Low	112 (72.3)	60 (77.9)	0.347
High	17 (11.0)	4 (5.2)	
N/A	26 (16.8)	13 (16.9)	
**iTIL group**			
Low	69 (44.5)	33 (42.9)	0.968
High	60 (38.7)	31 (40.3)	
N/A	26 (16.8)	13 (16.9)	
**Fibrosis**			
0-2	100 (64.5)	48 (62.3)	0.064
3	13 (8.4)	14 (18.2)	
N/A	42 (27.1)	15 (19.5)	

**Abbreviations:** BCS, Breast Conserving Surgery; FUSCC, Fudan University Shanghai Cancer Center; HRD, Homologous Recombination Deficiency; IQR, Interquartile Range; iTIL, Intratumoral Tumor-infiltrating Lymphocyte; LN, Lymph Node; MMR, Mismatch Repair; N/A, Not Available; sTIL, Stromal Tumor-infiltrating Lymphocyte.

## Abbreviations

TNBC: triple-negative breast cancer
SYTL4: synaptotagmin-like 4
FUSCC: Fudan University Shanghai Cancer Center
NAC: neoadjuvant chemotherapy
pCR: pathologic complete response
PD: progressed disease
SI: sensitization index
IHC: immunohistochemical
IC50: half maximal inhibitory concentration
HR: hazard ratio
DFS: disease-free survival
RFS: recurrence-free survival
qRT-PCR: quantitative real-time polymerase chain reaction
