# Supplementary material for: SYTL4 downregulates microtubule stability and confers paclitaxel resistance in triple-negative breast cancer
Source: Theranostics. 2020 Aug 29;10(24):10940–56. doi: 10.7150/thno.45207 (PMC7532662; doi:10.7150/thno.45207)
Supplement: Supplementary file 1 — Supplementary figures and tables. [file thnov10p10940s1.pdf]

Index

1. Supplementary Methods

2. Supplementary Tables

3. Supplementary Figures

1. Supplementary Methods

Standards for ranking genes

Briefly, each gene from the 434 merged candidate genes were scored according to a list of standards as follows:

1. Whether this gene can predict pCR in TNBC subgroup in any of these paclitaxel-based neoadjuvant chemotherapy datasets (GSE6861, GSE20271, GSE41998, GSE25066, GSE20194) or not?

1 = Yes in at least one dataset **0 =** No in any dataset

2. Whether this gene can define poor recurrence-free survival (RFS) in FUSCC TNBC cohort A [1]?

1 = High expression, poor RFS (log-rank $P < 0.1$) **0 =** Not correlated with poor RFS

3. Whether this gene can define poor RFS in KM-plotter TNBC cohort [2]?

1 = High expression, poor RFS (log-rank $P < 0.05$) **0 =** Not correlated with poor RFS

4. Whether this gene is upregulated in the paclitaxel-resistance cell originated from MDA-MB-231 cells in our lab?

1 = Upregulated (fold change > 1.2) **0 =** Not upregulated

Detailed scores for the top 30 genes are listed in Table S2.

siRNA pool procedure

A mixture of 50 μL of Opti-MEM (Invitrogen, USA), 0.3 μL of Lipofectamine RNAiMAX Transfection Reagent (Thermo Fisher Scientific, USA) and 50 nM individual siRNA was preincubated in triplicate in 96-well plates. Cells were seeded in 100 μL antibiotic-free

DMEM containing 10% FBS (3000 cells/well). This condition allowed a transfection efficiency greater than 95%, as validated by the uptake of a fluorescently tagged siRNA. After 24 h transfection, the medium was replaced with 200 μL either paclitaxel-containing medium (working concentration: 1.2 nM) or DMSO-containing medium for additional 72 h. The inhibition rate of cell proliferation was measured with a Cell Counting Kit-8 (CCK-8) (Dojindo, Japan) according to the manufacturer's protocol.

Correlation analysis between IC50 value and SYTL4 expression in public datasets

Gene expression data of breast cancer cell lines was obtained from Varley KE *et al*. (GSE58135) [3]. Values of half maximal inhibitory concentration (IC50) were extracted from Lawrence, R. T. *et al* [4]. The -$\log_{10}$IC50 (M) were plotted versus the relative mRNA expression of SYTL4 after log transformation for the five breast cancer cell lines. Pearson's correlation and Spearman's correlation R^2 with corresponding P values were determined.

Survival Analysis in Extended TNBC Cohorts

To determine whether SYTL4 mRNA expression were associated with patient survival and chemotherapy sensitivity, we first obtained a publicly available TNBC cohort from the Kaplan Meier-plotter [2] as the KM-plotter TNBC cohort (n = 126). Next, we analyzed a total of 232 TNBC patients who received chemotherapy in our center as FUSCC TNBC cohort B [5]. SYTL4 expression was stratified into low and high expression groups according to SYTL4 mRNA expression using an optimal cut-off. Finally, we defined SYTL4 protein expression into high and low groups based on

immunohistochemistry staining assay in who received chemotherapy in our center as FUSCC TNBC cohort C (n = 257). Survival analysis and a cox proportional hazard regression over SYTL4 expression status were performed within patients who received chemotherapy, patients who received taxane-containing chemotherapy, and patients who received non-taxane-containing chemotherapy. Recurrence-free survival (RFS) was defined as the time from surgery to recurrence (local, regional, or distant) or death from any cause. Overall survival (OS) was defined as the time from surgery to death from any cause [6].

Immunohistochemistry staining

Immunohistochemistry was performed and evaluated using a two-step method as described previously [7]. Immunostaining was performed on the TNBC cohort C using the tissue microarrays (TMAs). A rabbit polyclonal antibody against SYTL4 (Abcam, USA.) was applied to the TMAs. The TMAs were constructed as described previously using two tissue cores (1.0-mm diameter) taken from representative areas of each formalin-fixed, paraffin-embedded tumor specimen. The immunohistochemical staining of SYTL4 was mainly found in the cytoplasm of tumor cells. All spots were scored by two independent observers in a blinded fashion, and the observers did not have prior knowledge of the clinicopathological details of the samples. Discrepancies in scoring results between the two pathologists were resolved by discussion and consensus. For the quantification of SYTL4 expression, both the staining intensity and the percentage of stained cells were evaluated as previous reports [7]. The staining intensity was quantified on a 0-3 scale.

The cells with no staining were scored as 0 points, 1 point represented weak staining intensity, 2 points represented moderate staining intensity, and 3 points represented strong staining intensity. The percentage of reactive tumor cells was scored as follows: 0, less than 5%; 1, 5%-25%; 2, 26-50%; 3, 51%-75%; and 4, greater than 75%. The histological score (H-score) for SYTL4 expression in each spot was computed using the following formula: H-score = percentage score × intensity score. For each case, the corresponding H-score was calculated by averaging the H-scores of all the corresponding cores. For statistical analysis, H-scores (ranging from 0-12) of 0 to 7 were considered low expression and scores of 8 to12 considered high expression.

Synergistic effect calculation

An online drug synergism analysis tool SynergyFinder (version 2.0) (https://synergyfinder.fimm.fi/) was used to calculate the synergistic effect of the combination of paclitaxel and carboplatin. Detailed procedure was conducted according to guidelines on this web.

References

1. Liu YR, Jiang YZ, Xu XE et al. Comprehensive Transcriptome Profiling Reveals Multigene Signatures in Triple-Negative Breast Cancer. Clin Cancer Res 2016; 22: 1653-1662.
2. Nagy A, Lanczky A, Menyhart O, Gyorffy B. Validation of miRNA prognostic power in hepatocellular carcinoma using expression data of independent datasets. Sci Rep 2018; 8: 9227.
3. Varley KE, Gertz J, Roberts BS et al. Recurrent read-through fusion transcripts in breast cancer. Breast Cancer Res Treat 2014; 146: 287-297.
4. Lawrence RT, Perez EM, Hernandez D et al. The proteomic landscape of triple-negative breast cancer. Cell Rep 2015; 11: 630-644.
5. Jiang YZ, Ma D, Suo C et al. Genomic and Transcriptomic Landscape of Triple-Negative Breast Cancers: Subtypes and Treatment Strategies. Cancer Cell 2019; 35: 428-440 e425.
6. Gourgou-Bourgade S, Cameron D, Poortmans P et al. Guidelines for time-to-event end point definitions in breast cancer trials: results of the DATECAN initiative (Definition for the Assessment of Time-to-event Endpoints in CANcer trials)dagger. Ann Oncol 2015; 26: 873-879.
7. Zhao S, Liu XY, Jin X et al. Molecular portraits and trastuzumab responsiveness of estrogen receptor-positive, progesterone receptor-positive, and HER2-positive breast cancer. Theranostics 2019; 9: 4935-4945.
8. Ianevski A, Giri AK, Aittokallio T. SynergyFinder 2.0: visual analytics of multi-drug combination synergies. Nucleic Acids Res 2020; 48: W488-W493.

Table S1. Tumor and disease characteristics for sequenced TNBC patients

	Resistant group					Sensitive group		
	R1	R2	R3	R4	R5	S1	S2	S3
Age at diagnosis (years)	50	56	63	54	31	54	37	64
Neoadjuvant drugs	PC x 4	PC x 2	PC x 4	PC x 4	PC x 4	PC x 4	PC x 4	PC x 4
Response	SD	SD	SD	PD	PD	pCR	pCR	pCR
Sequenced samples	Pre	Pre	Pre	Pre & Post	Pre & Post	Pre	Pre	Pre
**Primary tumor**								
Ki-67	80%	80%	5%	NA	95%	80%	NA	70%
Tumor size (cm)	5	4	5	6	4	6.5	5.6	2
Lymph node status	cN0	cN0	cN0	cN2	cN0	cN1	cN1	cN2
**Post NAC pathology**								
pT	4cm	4cm	4cm	7.5cm	6cm	No residual invasive cancer	No residual invasive cancer	No residual invasive cancer
pN	pN0	pN0	pN0	NA	pN2	pN0	pN0	pN1
Ki-67	80%	70%	5%	10%	90%	/	/	/
CK5/6	+	-	-	-	-	/	/	/
CK14	-	+	-	-	-	/	/	/
E-cad	+	+	+	+	+	/	/	/
EGFR	+	-	+	-	+	/	/	/
**Adjuvant therapy**								
Surgery	M + ALND	M + ALND	M + ALND	M	M + ALND	M + ALND	M + ALND	M + ALND
Chemotherapy	PC x 1-FEC x 3	PC x 4	PC x 4	GP x 4	EC x 3	EC x 4	EC x 2	EC x 4

Radiotherapy	Yes	Yes	No	No	Yes	No	Yes	Yes
**Survival outcome**								
OS	Alive	Deceased	Alive	Deceased	Deceased	Alive	Alive	Alive
RFS	No event	Metastasis	No event	Metastasis	Metastasis	No event	No event	No event
OS time (months)	43.9	54.3	63.1	12.0	13.0	53.2	68.0	45.1
RFS time (months)	43.9	38.1	63.1	1.0	2.3	53.2	68.0	45.1

Abbreviations: ALND: axillary lymph node dissection; **RFS**: recurrence-free survival; **EC**: epirubicin + cyclophosphamide; **FEC**: fluorouracil + epirubicin + cyclophosphamide; **M**: mastectomy; **NA:** Not available; **NAC**: neo-adjuvant chemotherapy; **GP:** gemcitabine + cisplatin**; OS:** overall survival; **PC**: paclitaxel + carboplatin; **pCR**: pathologic complete response; **PD**: progressed disease; **pre:** pre-NAC; **post:** post-NAC; **SD**: stable disease; **TNBC**: triple-negative breast cancer.

Table S2. Differentially expressed genes across different neoadjuvant chemotherapy groups

	Top 100 upregulated genes
**pre-NAC resistant vs pre-NAC sensitive**	OSR1 CELSR2 NFASC KANK4 ANKRD18A TNS4 LGR6 ALDH1L1 TMEM59L CRISPLD1 SCN4A ANO1 BRSK2 ADCY5 ZNF483 FAM189A2 PRR36 PI16 FZD10 SFRP1 ATP6V1B1 KRT14 KLF5 TGFBR3 GPRIN2 LRRN2 MAGEE1 SIM1 GNAZ PIPSL STUM USP44 B3GNT4 AMIGO1 DGAT2 TMEM8B MPPED2 MFSD4A COL27A1 CLSTN2 C6orf25 ITGA2B IL17RD TNN MMP16 FAM184A L1TD1 ALX4 CLEC4F PTCHD1 ZNF462 PTPRZ1 SUSD5 COL20A1 HRCT1 RGMA XYLB MYH11 HPSE2 SYP HMGCLL1 PAK6 TUB TMEM139 PCDH19 GJB5 PEG3 SYT7 PITPNM3 B4GALNT4 TMEM130 ALDH3A1 PDZRN3 RIPPLY3 ANKRD36BP1 KLC3 ZNRF3 SPARCL1 KCNB1 ATP9A CRIM1 PDE9A CNTNAP3 EPHB3 CCDC120 SLC22A31 ITGB4 SOGA3 FBXW4P1 CBX3P2 FN3K CARMIL1 ZNF704 MAGI1 TCF7L2 SLC35F1 HACE1 ITGA6 NKD2 RELN
**post-NAC residual vs pre-NAC resistant**	CIDEC AKR1C2 ADIPOQ AQP7 HBG2 PTN GPD1 MAP1LC3C TUSC5 ATP1A2 PLIN4 FXYD1 HBG1 ITIH5 RBP4 TMEM132C PAMR1 PLIN1 HSPB6 ANGPTL1 MEST LIPE MAOA ACVR1C AKR1C3 EGF FABP4 MAMDC2 LEP PCK1 LYVE1 CDO1 AKR1C1 TNNT3 SDPR ADH1B LGALS12 CMYA5 SMYD1 HBA2 SCARA5 ADIRF TNNC1 SLC19A3 ITGA7 ACACB RERG ALAS2 IGFBP6 AOC3 PGM5 DNASE1L3 MEOX2 MYOM1 TNXA PKDCC PTGER3 PALM C12orf39 SPOCK3 DUSP1 PLP1 CHRDL1 SLC4A1 MFAP4 G0S2 CA3 TIMP4 HBB TNMD PDGFD KLF4 FOSB FLNC LDB3 PYGM MLXIPL FHL1 NOS1 GLYAT CFD PDK4 FOS EEF1A2 MMRN1 PER1 CASQ1 OGN C1QTNF7 CASQ2 HBA1 TRDN GHR GPAM DPT GPC3 RRAD CIDEA ABCA8 SCN7A

Abbreviations: NAC: neo-adjuvant chemotherapy.

Table S3. Standards of scoring genes

Gene	Protein	Predict pCR in neoadjuvant taxane-based chemotherapy datasets	Predict poor RFS in FUSCC TNBC cohort A (P < 0.1)	Predict poor RFS in KMplot TNBC cohort (P < 0.05)	Upregulated in paclitaxel-resistance cells	Sum
**AHR**	aryl hydrocarbon receptor	1	0	0	1	2
**AMOTL2**	angiomotin-like protein 2	1	0	0	1	2
**ARHGEF10**	rho guanine nucleotide exchange factor 10	1	0	0	1	2
**ATRNL1**	attractin-like-1	1	0	0	1	2
**BCAM**	basal cell adhesion molecule (Lutheran blood group)	1	0	1	1	3
**COL16A1**	collagen type XVI alpha 1 chain	1	0	0	1	2
**CPE**	carboxypeptidase E	1	0	0	1	2
**CTPS2**	CTP synthase 2	1	1	0	1	3
**EGFR**	epidermal growth factor receptor	1	0	1	0	2
**FGFR2**	fibroblast growth factor receptor 2	1	0	0	1	2
**FKBP9**	FKBP prolyl isomerase 9	1	0	1	1	3
**HMCN1**	hemicentin 1	1	0	1	0	2
**LAMA2**	laminin subunit alpha 2	1	0	0	1	2
**LAMA3**	laminin subunit alpha 3	1	0	1	1	3
**LRP6**	LDL receptor related protein 6	1	0	0	1	2

**MICAL3**	microtubule associated monooxygenase, calponin and LIM domain containing 3	1	0	0	1	2
**MID1**	midline 1	1	0	0	1	2
**PDGFRB**	platelet derived growth factor receptor beta	1	0	1	1	3
**PPP2R3A**	protein phosphatase 2 regulatory subunit B" Alpha	1	0	0	1	2
**PROS1**	protein S (alpha)	1	0	0	1	2
**PTPRS**	protein tyrosine phosphatase receptor type S	1	0	1	0	2
**SIK2**	salt inducible kinase 2	1	0	1	0	2
**SPTBN1**	spectrin beta, non-erythrocytic 1	1	0	1	0	2
**SYTL4**	synaptotagmin like 4	1	1	1	1	4
**TEAD1**	TEA domain transcription factor 1	1	0	1	0	2
**THRA**	thyroid hormone receptor alpha	1	0	1	1	3
**TNS1**	tensin 1	1	0	0	1	2
**TNXB**	tenascin XB	1	0	0	1	2
**ZHX3**	zinc fingers and homeoboxes protein 3	1	0	1	1	3
**ZNF160**	zinc finger protein 160	1	0	1	1	3

Abbreviations: NAC: neo-adjuvant chemotherapy; **pCR**: pathologic complete response; **RFS**: recurrence-free survival**;** **TNBC**: triple-negative breast cancer.

Table S4. Enriched hallmark gene sets of SYTL4 high and low expression group in TNBC cohort B from FUSCC by GSEA analysis (FDR q-val < 0.25)

Name	Size	NES	NOM p-val	FDR q-val
**Enriched in SYTL4 high group**				
HALLMARK_UV_RESPONSE_DN	142	1.96	< 0.001	0.10
HALLMARK_MYOGENESIS	184	1.87	< 0.001	0.10
HALLMARK_EPITHELIAL_MESENCHYMAL_TRANSITION	197	1.85	< 0.001	0.08
HALLMARK_APICAL_JUNCTION	191	1.78	0.02	0.11
HALLMARK_ANGIOGENESIS	33	1.70	0.01	0.15
HALLMARK_COAGULATION	116	1.63	0.04	0.20
HALLMARK_TGF_BETA_SIGNALING	53	1.63	0.06	0.18
HALLMARK_XENOBIOTIC_METABOLISM	178	1.53	0.05	0.23
**Enriched in SYTL4 low group**				
HALLMARK_MYC_TARGETS_V2	58	-2.01	< 0.001	0.02
HALLMARK_MYC_TARGETS_V1	195	-1.93	0.01	0.03
HALLMARK_UNFOLDED_PROTEIN_RESPONSE	112	-1.66	0.08	0.16
HALLMARK_E2F_TARGETS	195	-1.65	0.05	0.13
HALLMARK_G2M_CHECKPOINT	195	-1.60	0.09	0.14

Abbreviations: FDR: false discovery rate; **FUSCC**: Fudan University Shanghai Cancer Center; **GSEA**: Gene Set Enrichment Analysis; **NES**: normalized enrichment score; **SYTL4**: synaptotagmin-like 4; **NOM p-val:** nominal P value; **TNBC**: triple-negative breast cancer

Table S5. Top20 enriched KEGG pathways of SYTL4 high cluster in single-cell TNBC dataset

**Term**	**Count**	**Background number**	**Rich Factor**	**P Value**	**Corrected P Value**
Metabolic pathways	173	1433	0.12	2.60E-78	7.79E-76
Valine, leucine and isoleucine degradation	17	48	0.35	3.52E-15	5.28E-13
Peroxisome	20	83	0.24	6.66E-15	6.66E-13
Huntington disease	26	193	0.13	1.35E-13	9.96E-12
Thermogenesis	28	231	0.12	1.66E-13	9.96E-12
Oxidative phosphorylation	22	133	0.17	2.60E-13	1.30E-11
Protein processing in endoplasmic reticulum	23	165	0.14	1.82E-12	6.86E-11
Carbon metabolism	20	117	0.17	1.83E-12	6.86E-11
Parkinson disease	21	142	0.15	6.08E-12	2.03E-10
Non-alcoholic fatty liver disease (NAFLD)	20	149	0.13	9.20E-11	2.53E-09
FoxO signaling pathway	19	132	0.14	9.27E-11	2.53E-09
Alzheimer disease	21	171	0.12	1.40E-10	3.25E-09
Ribosome	20	153	0.13	1.41E-10	3.25E-09
Biosynthesis of amino acids	15	75	0.20	1.55E-10	3.28E-09
Insulin signaling pathway	19	137	0.14	1.64E-10	3.28E-09
Glutathione metabolism	12	56	0.21	5.37E-09	1.01E-07
Endocytosis	22	244	0.09	1.02E-08	1.80E-07
Vibrio cholerae infection	11	50	0.22	1.84E-08	3.07E-07
Citrate cycle (TCA cycle)	9	30	0.30	3.78E-08	5.98E-07
AMPK signaling pathway	15	120	0.13	4.74E-08	7.12E-07

Abbreviations: KEGG: Kyoto Encyclopedia of Genes and Genomes; **SYTL4**: synaptotagmin-like 4; **TNBC**: triple-negative breast cancer

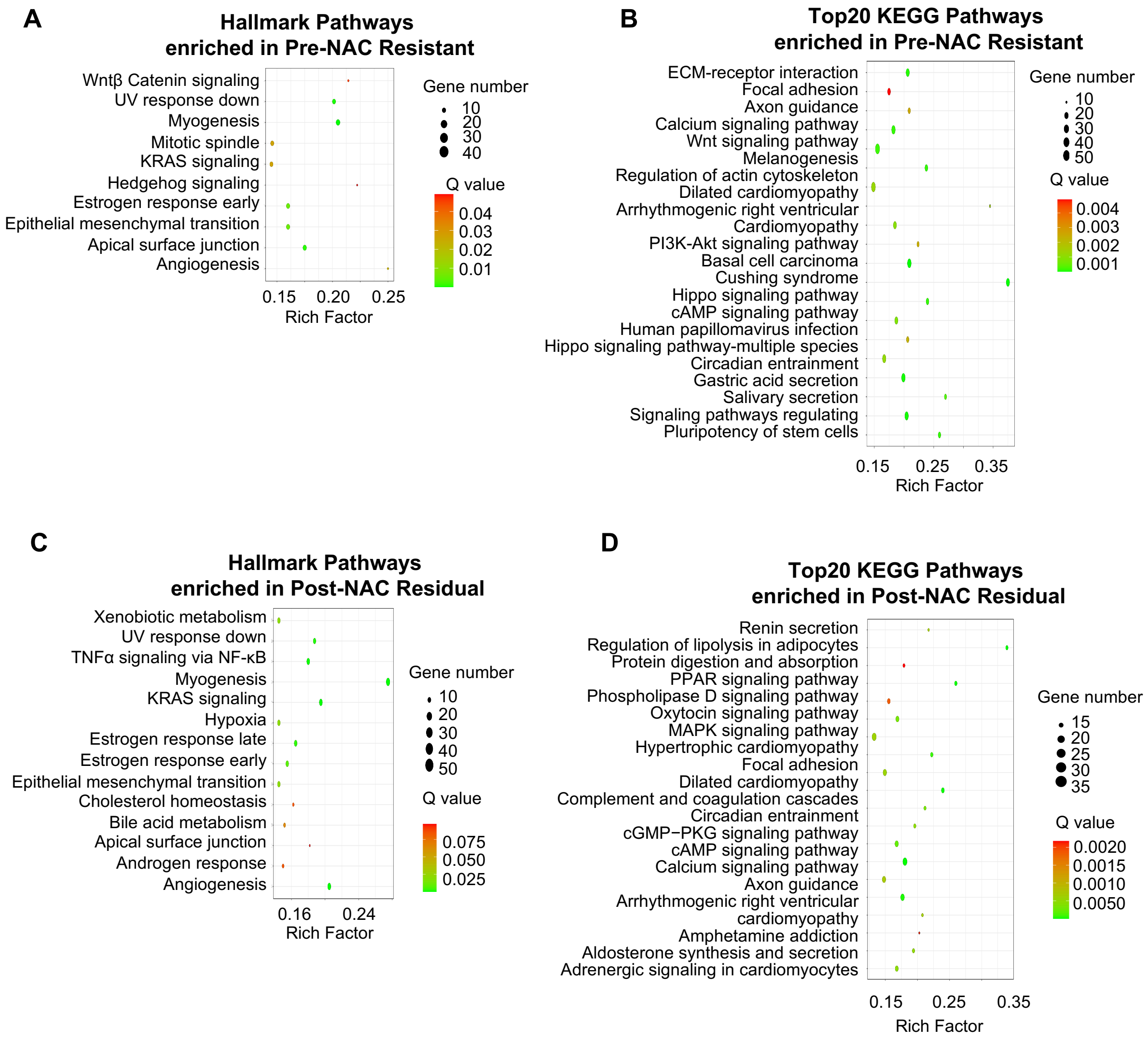

Figure S1. Enriched Hallmark and KEGG pathways.

(A) Enriched hallmark pathways and (B) top 20 KEGG pathways of 1,852 upregulated genes (fold change > 1, P < 0.05) from pre-NAC resistant samples compared with pre-NAC sensitive samples.

(C) Enriched hallmark pathways and (D) top 20 KEGG pathways of 1,253 upregulated genes (fold change > 1, P < 0.05) from post-NAC residual samples compared with pre-NAC resistant samples.

“Gene number” represents the number of called genes. “Rich factor” means the ratio of the number of called genes to the background number annotated in a certain pathway.

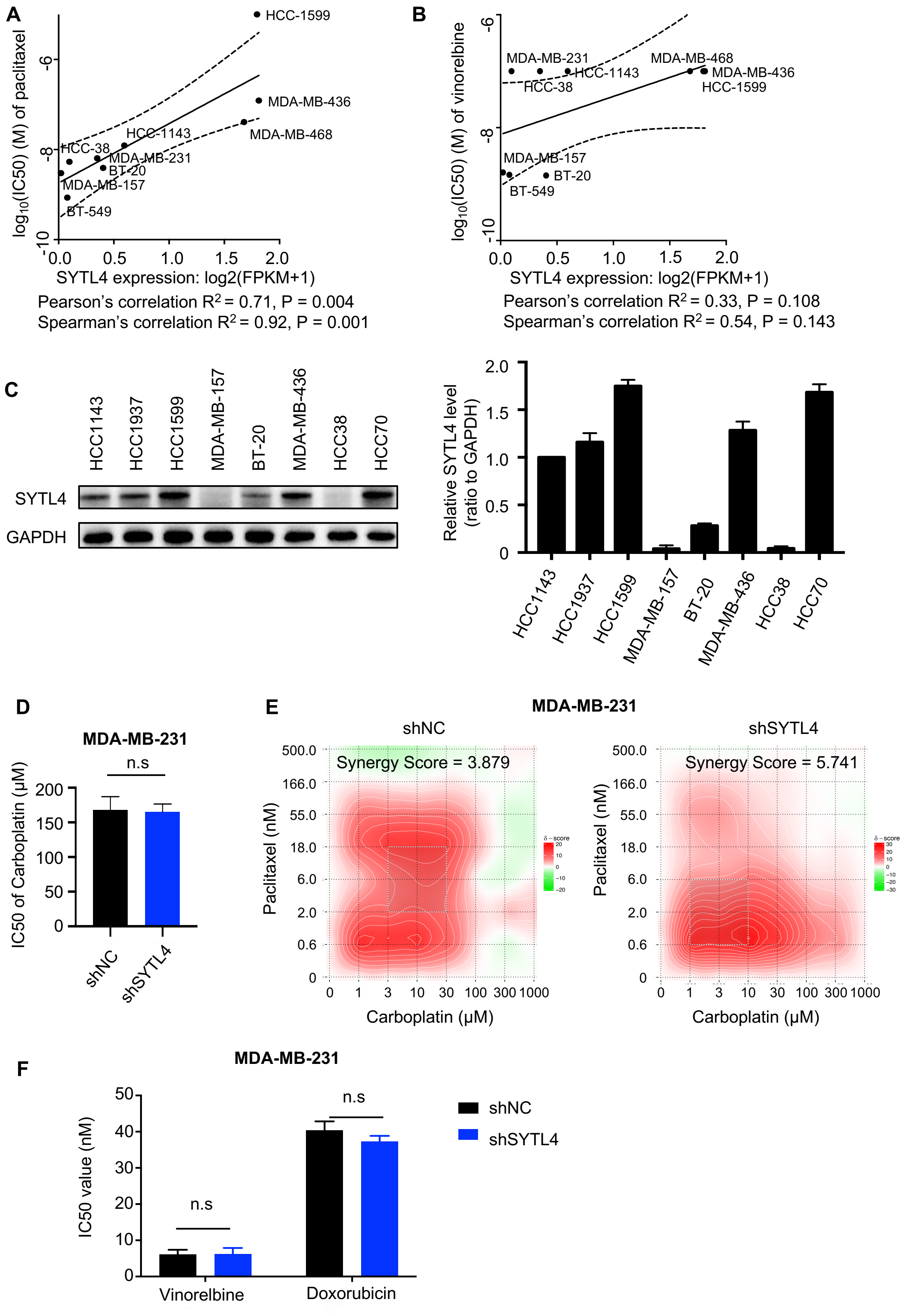
A
log10(IC50) (M) of paclitaxel
SYTL4 expression: log2(FPKM+1)
HCC-1599
MDA-MB-436
MDA-MB-468
HCC-1143
HCC-38
MDA-MB-231
BT-20
MDA-MB-157
BT-549
Pearson's correlation R2 = 0.71, P = 0.004
Spearman's correlation R2 = 0.92, P = 0.001
B
log10(IC50) (M) of vinorelbine
SYTL4 expression: log2(FPKM+1)
MDA-MB-231
HCC-1143
HCC-38
MDA-MB-468
MDA-MB-436
HCC-1599
MDA-MB-157
BT-20
BT-549
Pearson's correlation R2 = 0.33, P = 0.108
Spearman's correlation R2 = 0.54, P = 0.143
C
HCC1143
HCC1937
HCC1599
MDA-MB-157
BT-20
MDA-MB-436
HCC38
HCC70
SYTL4
GAPDH
Relative SYTL4 level (ratio to GAPDH)
D
MDA-MB-231
n.s
IC50 of Carboplatin (μM)
shNC
shSYTL4
E
MDA-MB-231
shNC
shSYTL4
Synergy Score = 3.879
Synergy Score = 5.741
Paclitaxel (nM)
Carboplatin (μM)
δ-score
F
MDA-MB-231
n.s
IC50 value (nM)
Vinorelbine
Doxorubicin
shNC
shSYTL4

Figure S2. Sensitivity of MDA-MB-231 cells to paclitaxel, carboplatin, vinorelbine and doxorubicin.

Correlation between SYTL4 mRNA expression and IC50 of (A) paclitaxel or (B) vinorelbine in TNBC cells using public data. SYTL4 expression level was obtained from Varley KE et al. (GSE58135) [3]. Drug resistance values (IC50) were extracted from Lawrence, R. T. *et al* [4]. Pearson’s correlation and Spearman’s correlation were calculated and tested.

(C) Representative graph of western blot across TNBC cell lines (left). Quantified relative SYTL4 expression (relative to GAPDH) were normalized to HCC1143 (right) (mean ± SD, n = 3 independent experiments).

(D) IC50 of carboplatin in MDA-MB-231 cells expressing shNC or shSYTL4 (mean ± SD, n = 3 independent experiments, unpaired t test).

(E) Addictive effect of the combination of paclitaxel and carboplatin in MDA-MB-231 cells. Left panel: shNC; Right panel: shSYTL4. Synergy Score was calculated through on-line tool “SynergyFinder 2.0” [8]. Synergy Score < 10 indicates addictive effect.

(F) IC50 of vinorelbine and doxorubicin in MDA-MB-231 cells expressing shNC or shSYTL4 (mean ± SD, n = 3, unpaired t test).

* P < 0.05; ** P < 0.01; *** P < 0.001; n.s: not significant.

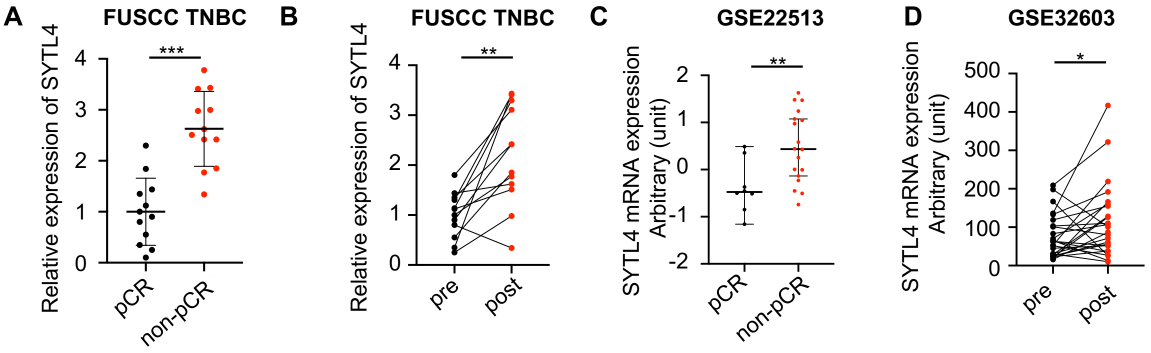

Figure S3. SYTL4 expression in breast tumors before and after neoadjuvant chemotherapy (NAC).

(A) Relative SYTL4 mRNA expression by qRT-PCR in pre-NAC biopsy tissues from pathological complete response (pCR) (n = 12) and non-pCR (n = 12) patients with triple-negative breast cancer (TNBC) who underwent taxane-based NAC in our center (mean ± SD, unpaired t test).

(B) Relative SYTL4 mRNA expression by qRT-PCR in paired pre- and post-NAC tissues from non-pCR patients with TNBC (n = 12) who underwent taxane-based NAC in our center (paired t test).

SYTL4 mRNA expression in pCR and non-pCR patients with breast cancer from (C) GSE22513, which included tumor tissues from pretreatment needle biopsies from breast cancer patients enrolled on a paclitaxel/radiation clinical trial (error bars: median with 95% CI, Mann-Whitney test).

(D) SYTL4 mRNA expression in pre- and post-NAC samples from GSE32603, which serially sequenced gene expression arrays in locally advanced breast cancer patients who failed to respond to neoadjuvant chemotherapy. Pretreatment biopsy were compared

to those biopsy specimens obtained in tumors surgically removed after chemotherapy (Wilcoxon matched-pairs signed rank test).

* $P < 0.05$; ** $P < 0.01$; *** $P < 0.001$; n.s: not significant.

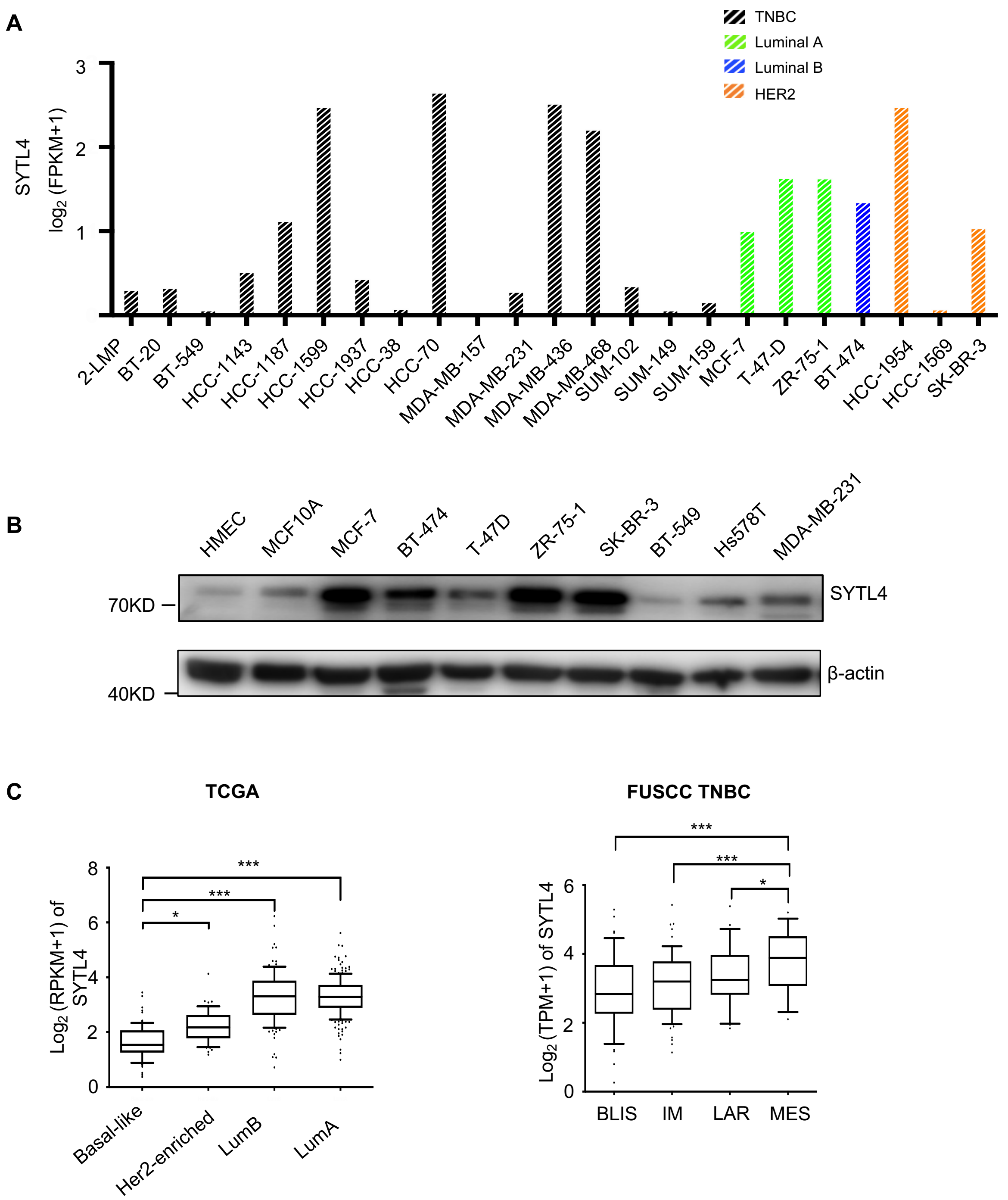

Figure S4. SYTL4 expression in breast cancer cell lines and tumor tissues.

(A) SYTL4 mRNA expression in breast cancer cell lines of different subtypes using data from GSE58135.

(B) SYTL4 protein expression in breast cancer cell lines by western blot.

(C) SYTL4 mRNA expression across (A) PAM50 subtypes of breast cancer from The Cancer Genome Atlas (TCGA) cohort and across (B) TNBC subtypes of from Fudan University Shanghai Cancer Center (FUSCC) cohort.

BLIS: basal-like immune-suppressed; IM: immunomodulatory; LAR: luminal androgen receptor; Lum A: luminal A subtype; Lum B: luminal B subtype; MES: mesenchymal; TNBC: triple-negative breast cancer. (median with 90% CI, Kruskal-Wallis test).

* $P < 0.05$; ** $P < 0.01$; *** P <. 0.001; n.s: not significant.

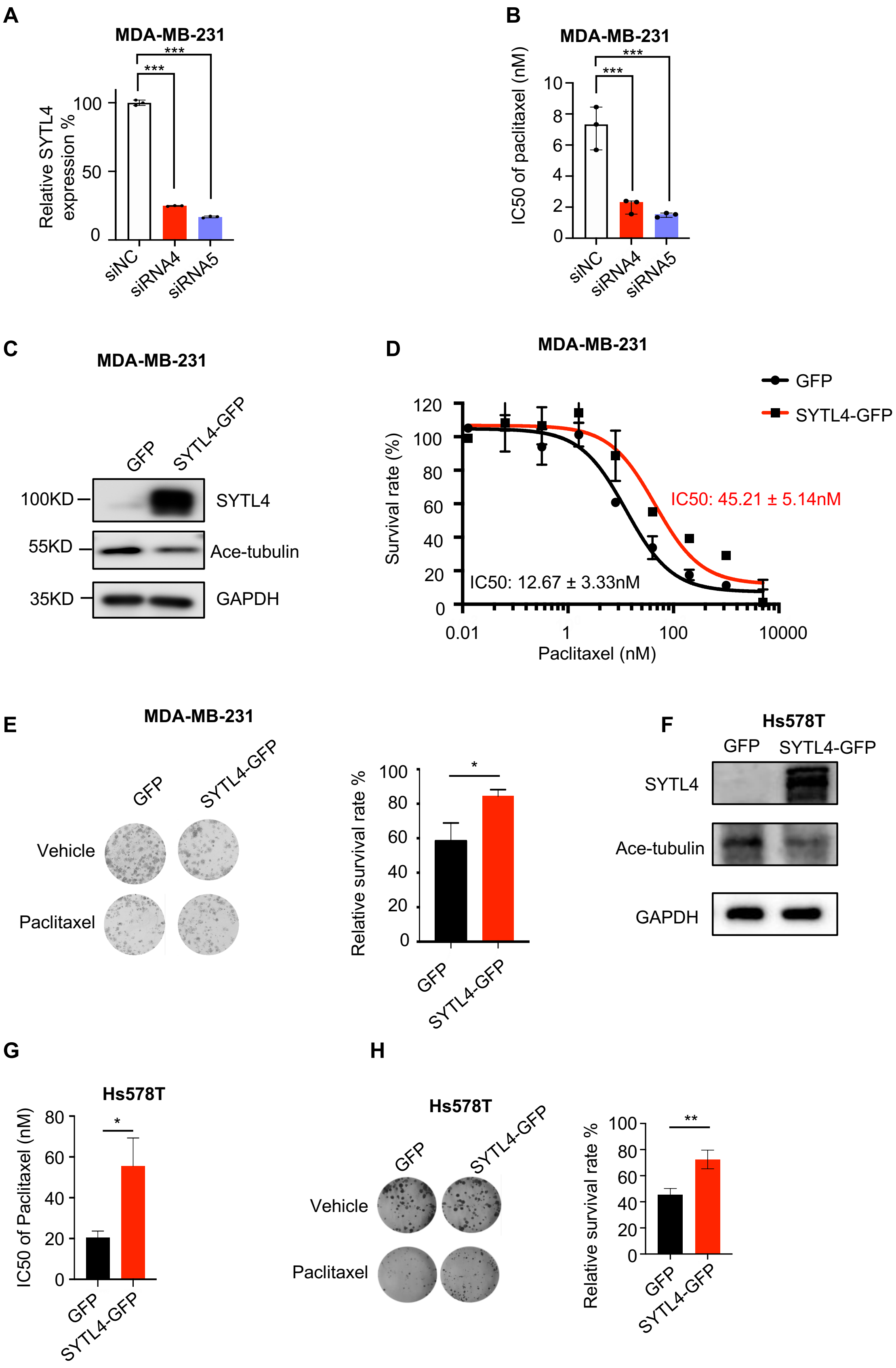
A
MDA-MB-231
***
***
Relative SYTL4 expression %
100
50
0
siNC
siRNA4
siRNA5
B
MDA-MB-231
***
***
IC50 of paclitaxel (nM)
10
8
6
4
2
0
siNC
siRNA4
siRNA5
C
MDA-MB-231
GFP
SYTL4-GFP
100KD
SYTL4
55KD
Ace-tubulin
35KD
GAPDH
D
MDA-MB-231
GFP
SYTL4-GFP
Survival rate (%)
120
100
80
60
40
20
0
IC50: 45.21 ± 5.14nM
IC50: 12.67 ± 3.33nM
0.01
1
100
10000
Paclitaxel (nM)
E
MDA-MB-231
GFP
SYTL4-GFP
Vehicle
Paclitaxel
Relative survival rate %
100
80
60
40
20
0
*
GFP
SYTL4-GFP
F
Hs578T
GFP
SYTL4-GFP
SYTL4
Ace-tubulin
GAPDH
G
Hs578T
IC50 of Paclitaxel (nM)
80
60
40
20
0
*
GFP
SYTL4-GFP
H
Hs578T
GFP
SYTL4-GFP
Vehicle
Paclitaxel
Relative survival rate %
100
80
60
40
20
0
**
GFP
SYTL4-GFP

Figure S5. Paclitaxel sensitivity and ace-tubulin level in MDA-MB-231 and Hs578Tcells.

(A) Relative SYTL4 expression normalized to siNC in MDA-MB-231 cells (mean ± SD, n = 3, one-way ANOVA test).

(B) IC50 of paclitaxel in MDA-MB-231 cells transfected by siNC, siRNA4, and siRNA5 (mean ± SD, n = 3 independent assays, one-way ANOVA test).

(C) Western blot of SYTL4-GFP overexpression in MDA-MB-231 cells.

(D) IC50 of paclitaxel in MDA-MB-231 with SYTL4-GFP overexpression compared to its GFP control.

(E) Cell colony formation in MDA-MB-231 cells treated with vehicle or paclitaxel. Relative cell survival rate in colony formation assay (left) was calculated by dividing the cell colony numbers with paclitaxel by colony numbers under vehicle treatment (right) (mean ± SD, n = 3, unpaired t test).

(F) Western blot analysis of Hs578T cells with SYTL4-GFP overexpression.

(G) IC50 of paclitaxel in Hs578T with SYTL4 overexpression (mean ± SD, n = 3, unpaired t test).

(H) Cell colony formation in Hs578T cells treated with vehicle or paclitaxel. Relative survival rate in colony formation assay (left) was calculated by dividing the cell colony numbers with paclitaxel by colony numbers under vehicle treatment (right) (mean ± SD, n = 3, unpaired t test).

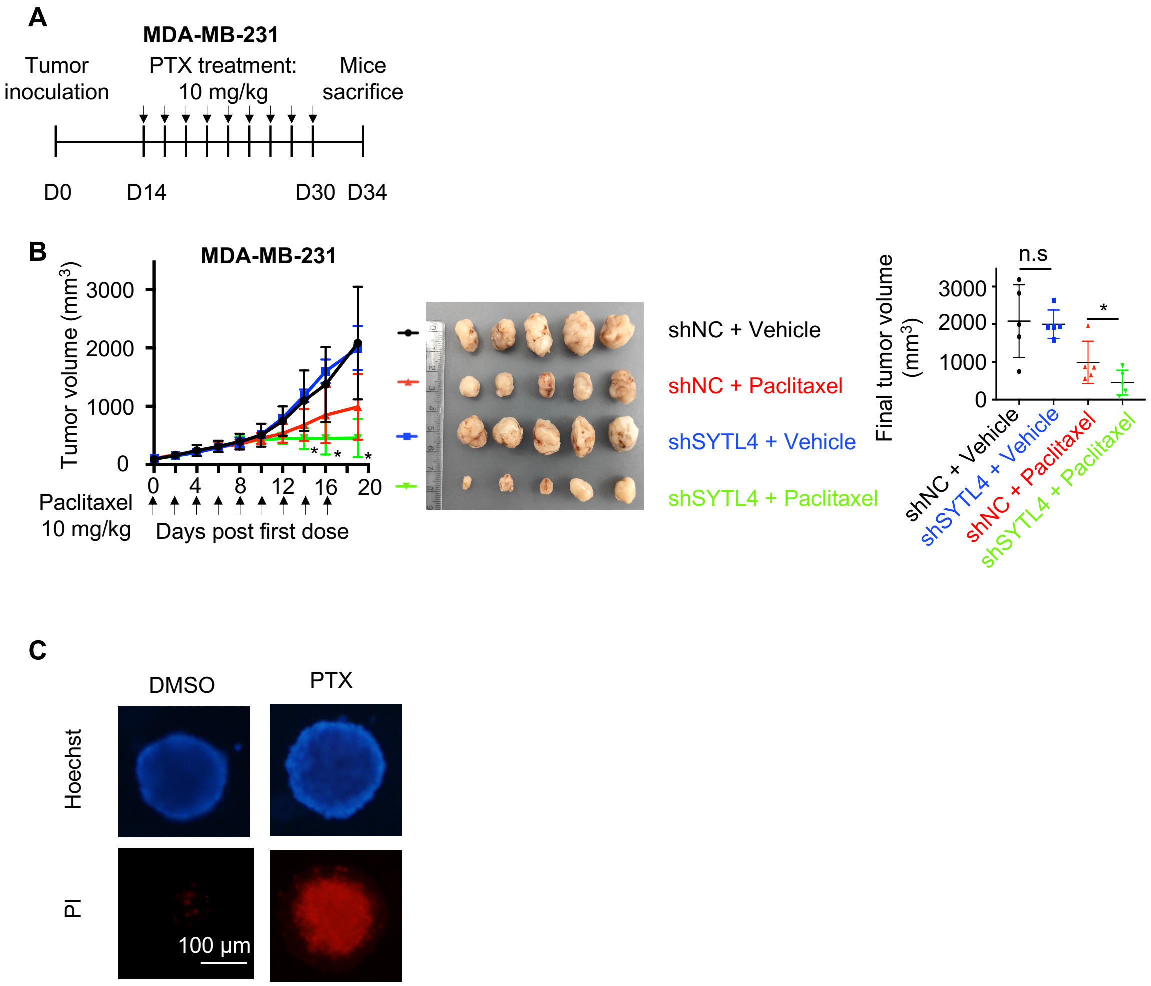

Figure S6. *In vivo* tumor growth and patient-derived organoids (PDO).

(A) Tumor growth and drug procedure. Cells were inoculated into BALB/c nude mice on D0, randomized when tumor size reached to 50-100 mm^3, treated with paclitaxel (PTX, 10 mg/kg) intraperitoneally every two day, and mice were sacrificed to resect tumors.

(B) SYTL4 knockdown inhibited tumor formation in nude mice after sequential paclitaxel treatment. MDA-MB-231 cells expressing shNC or shSYTL4 were transplanted into nude mouse mammary fat pads as described in the Methods section. *In vivo* growth curves quantified by tumor volume were illustrated (left). Arrows represent paclitaxel (10

mg/kg) treatment of tumor-bearing mice. A paired t test was conducted between the shSYTL4 + Paclitaxel group and the shNC + Paclitaxel group (mean ± SD, n = 5). Final tumor images were shown (middle). Final tumor volume was calculated (right) (mean ± SD, n = 5).

(C) Hoechst/PI staining of PDO with paclitaxel. Blue: Hoechst; Red: PI; PTX: paclitaxel.

* P < 0.05; ** P < 0.01; *** P < 0.001; n.s: not significant.

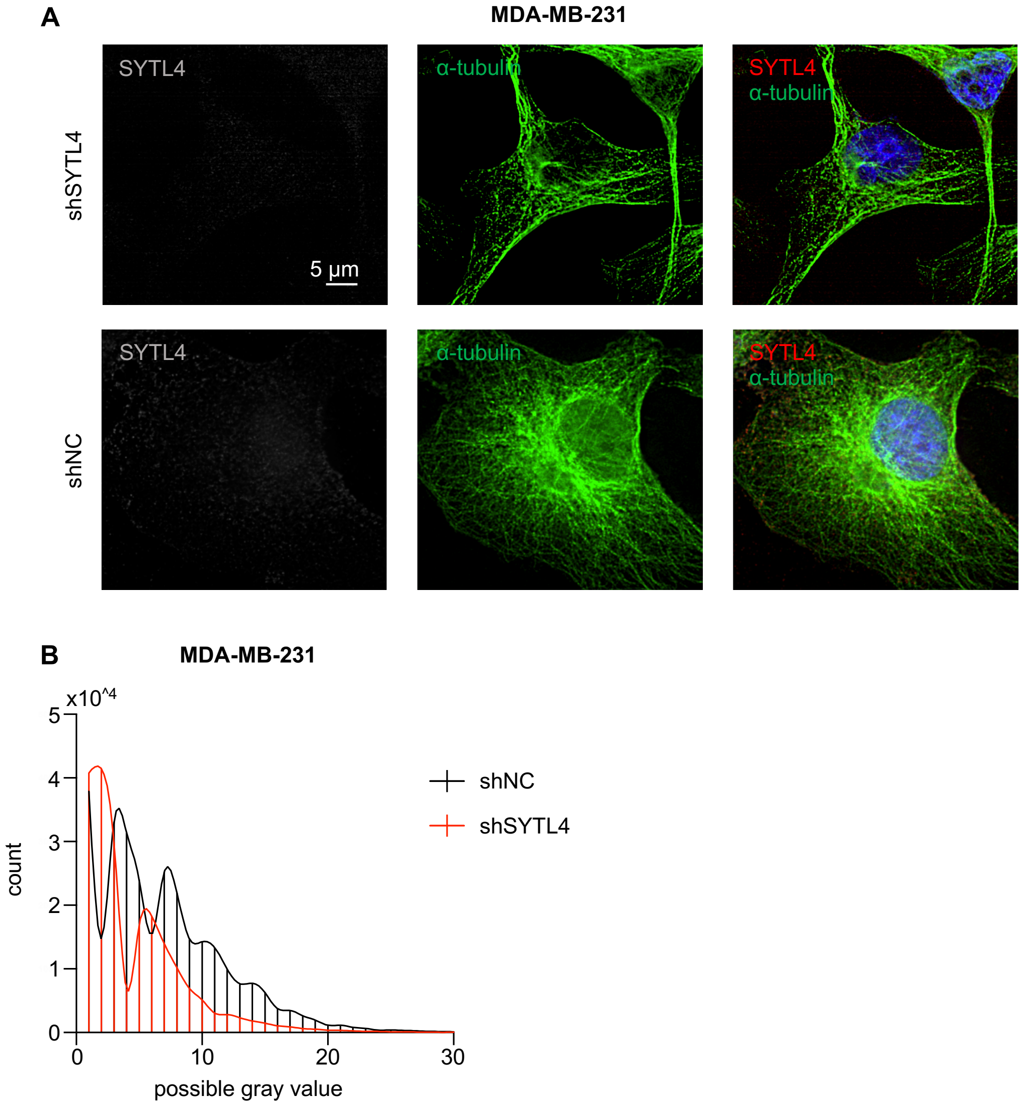

Figure S7. Immunofluorescent (IF) intensity of SYTL4 in MDA-MB-231 cells expressing shNC or shSYTL4.

(A) Representative IF graph of MDA-MB-231 cells. Gray: SYTL4; Green: α-tubulin; blue: DAPI.

(B) Histogram of gray values of SYTL4. Possible gray values of SYTL4 were measured by Fiji.

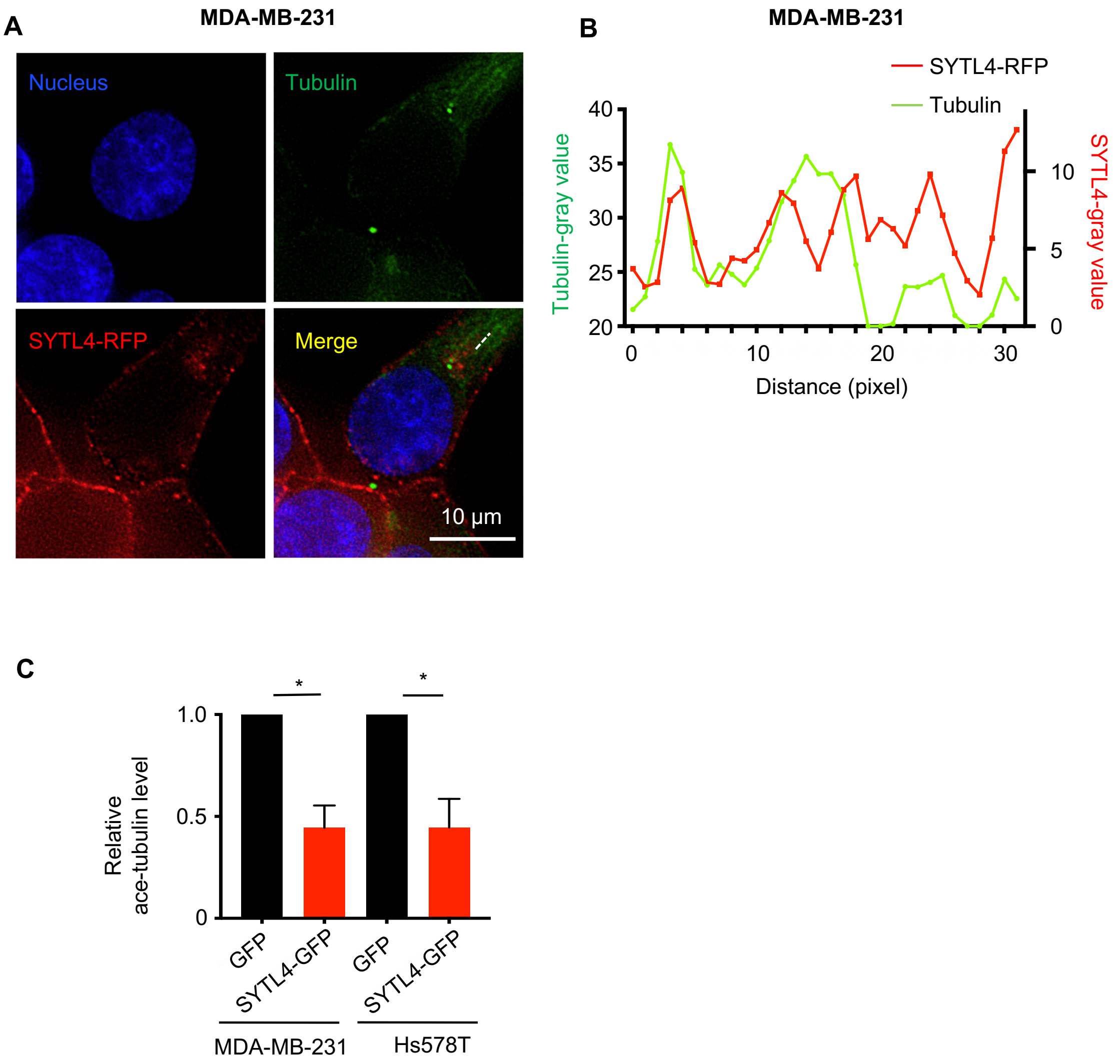

Figure S8. Live imaging of the localization of SYTL4 in SYTL4-RFP overexpressed MDA-MB-231 cells.

(A) Representative figure. MDA-MB-231 cells expressing SYTL4-RFP (red) were visualized using live imaging platform.

(B) Colocalization analysis of SYTL4 and microtubules. The intensity of green/red fluorescence along the dotted line was evaluated by Fiji.

(C) Relative ace-tubulin level in MDA-MB-231 and Hs578T cells expressing GFP or SYTL4-GFP. The intensity of ace-tubulin band was normalized to the baseline intensity level of its own GFP control. (mean ± SD, n = 3, one-way ANOVA test)

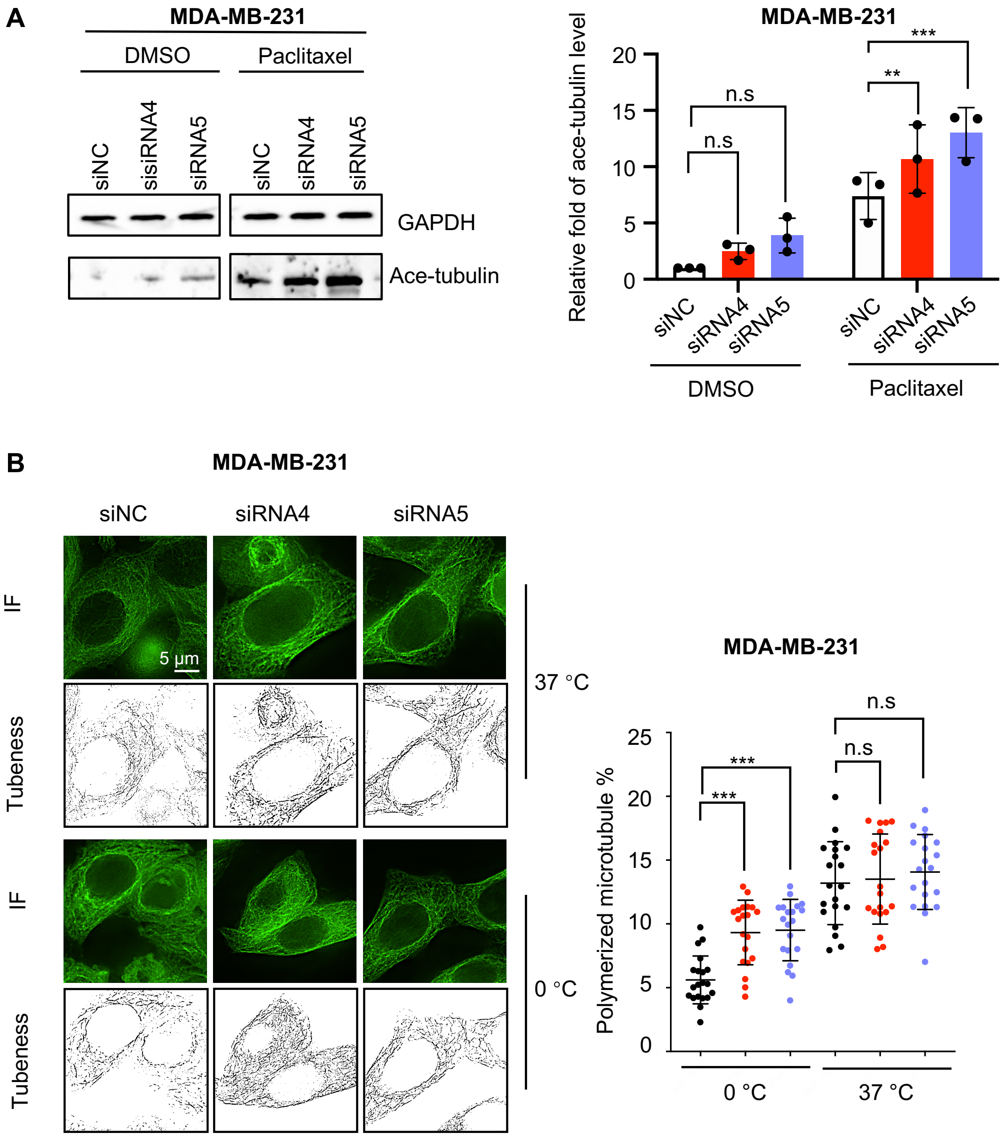

Figure S9. Microtubule acetylation level and microtubule network in MDA-MB-231 cells.

(A) Western blot analysis of microtubule acetylation in MDA-MB-231 cells transfected with siNC, siRNA4 and siRNA5 (left). Band intensity was estimated by Fiji. (B) Data represents

the band intensity of ace-tubulin relative to the baseline intensity level of siNC cells under DMSO treatment (mean ± SD, n = 3, one-way ANOVA test).

(B) Immunofluorescence analysis of microtubule network in MDA-MB-231 cells at 0 °C and 37 °C (left). Cells were fixed by 100% cold methanol. Tubule-like structure was revealed by Fiji using the Tubeness plugin. The percentage (%) of polymerized microtubules represented the ratio of the area of tubule-like structures to the region inside the cell contour (right) (mean ± SD, n = 20, one-way ANOVA test).
